# Cardiopulmonary resuscitation (CPR) during spaceflight - a guideline for CPR in microgravity from the German Society of Aerospace Medicine (DGLRM) and the European Society of Aerospace Medicine Space Medicine Group (ESAM-SMG)

**DOI:** 10.1186/s13049-020-00793-y

**Published:** 2020-11-02

**Authors:** Jochen Hinkelbein, Steffen Kerkhoff, Christoph Adler, Anton Ahlbäck, Stefan Braunecker, Daniel Burgard, Fabrizio Cirillo, Edoardo De Robertis, Eckard Glaser, Theresa K. Haidl, Pete Hodkinson, Ivan Zefiro Iovino, Stefanie Jansen, Kolaparambil Varghese Lydia Johnson, Saskia Jünger, Matthieu Komorowski, Marion Leary, Christina Mackaill, Alexander Nagrebetsky, Christopher Neuhaus, Lucas Rehnberg, Giovanni Marco Romano, Thais Russomano, Jan Schmitz, Oliver Spelten, Clément Starck, Seamus Thierry, Rochelle Velho, Tobias Warnecke

**Affiliations:** 1 German Society of Aviation and Space Medicine (DGLRM), Munich, Germany; 2 Department of Anaesthesiology and Intensive Care Medicine, University Hospital of Cologne, 50937 Cologne, Germany; 3 Space Medicine Group, European Society of Aerospace Medicine (ESAM), Cologne, Germany; 4 Department of Internal Medicine III, Heart Centre of the University of Cologne, Cologne, Germany; 5 Fire Department City of Cologne, Institute for Security Science and Rescue Technology, Cologne, Germany; 6 Department of Anaesthesia and Intensive Care, Örebro University Hospital, Örebro, Sweden; 7 Department of Anesthesiology, University of Florida College of Medicine, Jacksonville, FL USA; 8 Department of Cardiology and Angiology, Heart Center Duisburg, Evangelisches Klinikum Niederrhein, Duisburg, Germany; 9 Department of Anaesthesia and Intensive Care, Santa Maria delle Grazie Hospital, Pozzuoli, Naples, Italy; 10 Division of Anaesthesia, Analgesia, and Intensive Care, Department of Surgical and Biomedical Sciences, University of Perugia, Perugia, Italy; 11 Gerbrunn, Germany; 12 Department of Psychiatry and Psychotherapy, Faculty of Medicine and University Hospital Cologne, University of Cologne, 50937 Cologne, Germany; 13 Aerospace Medicine, Centre of Human and Applied Physiological Sciences, King’s College, London, UK; 14 Department of Otorhinolaryngology, Head and Neck Surgery, University of Cologne, 50937 Cologne, Germany; 15 University of Perugia-Terni, Perugia-Terni, Italy; 16 Cologne Center for Ethics, Rights, Economics, and Social Sciences of Health (CERES), University of Cologne and University Hospital of Cologne, Cologne, Germany; 17 Department of Surgery and Cancer, Faculty of Medicine, Imperial College London, Exhibition road, London, SW7 2AZ UK; 18 School of Nursing, University of Pennsylvania, Philadelphia, PA USA; 19 Accident and Emergency Department, Queen Elizabeth University Hospital, Glasgow, Scotland; 20 Department of Anesthesia, Critical Care and Pain Medicine, Massachusetts General Hospital, Harvard Medical School, Boston, USA; 21 Department of Anesthesiology, Heidelberg University Hospital, Heidelberg, Germany; 22 University Hospital Southampton NHS Foundation Trust, Anaesthetic Department, Southampton, UK; 23 Anesthesia and Postoperative Intensive Care Unit, AORN Cardarelli, Naples, Italy; 24 Centre of Human and Applied Physiological Sciences, Kings College London, London, UK; 25 Department of Anaesthesiology and Intensive Care Medicine, Schön Klinik Düsseldorf, Am Heerdter Krankenhaus 2, 40549 Düsseldorf, Germany; 26 Anesthesiology Department, Brest University Hospital, Brest, France; 27 Anesthesiology Department, Bretagne Sud General Hospital, Lorient, France; 28 Medical and Maritime Simulation Center, Lorient, France; 29 Laboratory of Psychology, Cognition, Communication and Behavior, University of Bretagne Sud, Vannes, France; 30 Academic Department of Anaesthesia, Critical Care, Pain and Resuscitation, University Hospitals Birmingham, Heart of England NHS Foundation Trust, Birmingham, UK; 31 University Department for Anesthesia, Intensive and Emergency Medicine and Pain Management, Hospital Oldenburg, Oldenburg, Germany

## Abstract

**Background:**

With the “Artemis”-mission mankind will return to the Moon by 2024. Prolonged periods in space will not only present physical and psychological challenges to the astronauts, but also pose risks concerning the medical treatment capabilities of the crew. So far, no guideline exists for the treatment of severe medical emergencies in microgravity. We, as a international group of researchers related to the field of aerospace medicine and critical care, took on the challenge and developed a an evidence-based guideline for the arguably most severe medical emergency – cardiac arrest.

**Methods:**

After the creation of said international group, PICO questions regarding the topic cardiopulmonary resuscitation in microgravity were developed to guide the systematic literature research. Afterwards a precise search strategy was compiled which was then applied to “MEDLINE”. Four thousand one hundred sixty-five findings were retrieved and consecutively screened by at least 2 reviewers. This led to 88 original publications that were acquired in full-text version and then critically appraised using the GRADE methodology. Those studies formed to basis for the guideline recommendations that were designed by at least 2 experts on the given field. Afterwards those recommendations were subject to a consensus finding process according to the DELPHI-methodology.

**Results:**

We recommend a differentiated approach to CPR in microgravity with a division into basic life support (BLS) and advanced life support (ALS) similar to the Earth-based guidelines. In immediate BLS, the chest compression method of choice is the Evetts-Russomano method (ER), whereas in an ALS scenario, with the patient being restrained on the Crew Medical Restraint System, the handstand method (HS) should be applied. Airway management should only be performed if at least two rescuers are present and the patient has been restrained. A supraglottic airway device should be used for airway management where crew members untrained in tracheal intubation (TI) are involved.

**Discussion:**

CPR in microgravity is feasible and should be applied according to the Earth-based guidelines of the AHA/ERC in relation to fundamental statements, like urgent recognition and action, focus on high-quality chest compressions, compression depth and compression-ventilation ratio. However, the special circumstances presented by microgravity and spaceflight must be considered concerning central points such as rescuer position and methods for the performance of chest compressions, airway management and defibrillation.

**Supplementary information:**

**Supplementary information** accompanies this paper at 10.1186/s13049-020-00793-y.

## Introduction

Manned spaceflight to the Moon or Mars [1] may become reality in the near future. In these exploration missions, both duration and medical requirements differ significantly from spaceflight in low earth orbit (LEO) [2, 3] and dictate the need for extensive planning for the management of life-threatening medical conditions. In contrast to LEO missions, evacuation during flights to the Moon or Mars will be challenging if not impossible [2, 4]. Although every crew member receives basic medical training and each crew has at least one Crew Medical Officer (CMO) with extended medical skills, the crew’s most medically trained member might become incapacitated. This will significantly limit the crew’s medical emergency treatment capabilities.

Given that a single medical emergency could endanger the whole mission, it is of utmost importance to develop medical procedures and guidelines to facilitate medical treatment in the remote environment. Based on an incidence of medical emergencies in the general population of 0.06 events per person per year, an astronaut crew of six having a flight duration of 2.74 years (or 900 days, the expected duration of a mission to Mars) may encounter an estimated 0.99 medical emergencies [5]. However, new calculations adopting the Integrated Medical Model estimate the incidence of crewmember incapacitation requiring evacuation during LEO mission as low as 0.017 events per person per year [6].

Cardiac arrest is a critical medical condition with high expected morbidity and mortality. It can have a variety of causes (e.g., cardiac event or injury, hypoxia, circulatory shock, toxic exposure, electric shock, or trauma) all of which remain possible during spaceflight [7]. Without immediate and effective cardiopulmonary resuscitation (CPR), cardiac arrest will result in the death of the crewmember. Since immediate, advanced and highly intensive management is required, cardiac arrest is likely to have profound effects on crew and has the potential to endanger the whole mission.

With its beginning in the 1950s and 60s with Kouwenhoven, Jude, and Knickerbocker [8, 9], research activities for CPR on Earth have been extensive. CPR guidelines from the American Heart Assosciation (AHA) and the European Resuscitation Council (ERC) are updated regularly with evolving evidence and have been adapted to a variety of provider and patient populations over the years. Although the current guidelines include a section on CPR in special circumstances [10], they do not address cardiac arrest management in microgravity. Furthermore, while CPR on Earth is a common occurence [11], it has, so far, never been practiced in reality on a human during spaceflight.

CPR represents a significant challenge even to trained health care professionals on Earth [12]. However, microgravity, limited physical space, and limitations in manpower and equipment are likely to complicate CPR during space missions. Adaptation of CPR guidelines to a microgravity environment exemplifies the fundamental challenges of CPR.

### Aims and scope of the guideline

The microgravity environment significantly complicates the delivery of CPR. Therefore, terrestrial guidelines cannot be used during spaceflight without several modifications and specifications. The aim of this guideline is to address the unique challenges of CPR in microgravity by reviewing, analyzing, and rating available scientific evidence. For specific topics where no or minimal previous research is available, expert opinion and consensus were used to generate recommendations.

## Material and methods

### Literature search strategy

A task force with 27 members was created by the ESAM-SMG and the DGLRM and consisted of a broad range of both medical and scientific experts that included Aerospace Medicine, pre-hospital and in-hospital Emergency Medicine, General Surgery, Cardiology, Anesthesiology, Intensive Care Medicine, and Physics. This task force then prepared a first list consisting of the essential steps of CPR that would need to be addressed in a guideline for the special environment of microgravity or spaceflight.

In total, there were 15 key-words which formed the basis for the creation of specific questions to guide the systematic literature research (Table 1). Those questions were then drafted utilizing the PICO-structure (Population, Intervention, Comparison, Outcome) [13, 14]. Every member was able to draft and propose keywords and PICO-questions and a consensus was built. Subsequently, the generated keywords and questions were reviewed and, if necessary, modified by the other task force members. Altogether, 134 questions were defined. Additional questions could not be structured using the PICO format (e.g., ethical questions).

**Table 1:** Relevant topics for the development of a guideline for CPR during spaceflight

1. Chest compressions	2. Automated chest compression devices	3. Airway management
4. Ventilation	5. Suction	6. Defibrillation
7.Intravenous access	8. Medication	9. Medical Training
10. ROSC	11. Death	12. Telemedicine
13. Reversible causes of cardiac arrest	14.Technical limitations of spaceflight	15. Ethics

For each of the 134 questions, a specific search-strategy for PubMed (“MEDLINE”; https://www.ncbi.nlm.nih.gov/pubmed/) was then created. In 75 cases, the questions did not differ in their search-strategy which led to a total of 59 different search strings, all listed in Additional file 1. The search strategy resulted in 4165 articles in PubMed.

The identified abstracts were inserted into the browser-based program “abstrackr” (http://abstrackr.cebm.brown.edu) to enable a time-effective screening process of the abstracts [15]. “Abstrackr” allows the reviewer to read the abstract and decide whether it seems relevant, irrelevant or of unknown relevance. Subsequently, every abstract was screened independently by two blinded reviewers.

In cases without conflicts, the abstract was either categorized as relevant or irrelevant. Conflicts were solved by the Chairperson after the review of the full-length paper. After the screening process, 432 abstracts were left of which 269 remained after removal of duplicates. Subsequently, the original papers were acquired using primarily “MEDLINE” and “Google Scholar”. This led to 88 available original papers, as the remaining 181 articles were either not written in the English language or, despite all efforts, not accessible.

### Quality of evidence

All 88 publications were analyzed using the GRADE methodology (The Grading of Recommendations Assessment, Development and Evaluation) to assess the quality of the retrieved literature based on study design limitations (selection, performance, detection, attrition and reporting bias), effect consistency and size, directness, precision, publication bias, dose-response effect and presence of antagonistic bias [16–19]. Quality of evidence was rated as either “high”, “moderate”, “low”, or “very low”.

Subsequently, the task force members were split into teams of two to tackle one of the 15 main topics. Every task force member was part of two teams in total and was asked to use the original scientific papers, acquired through the systematic literature search, to develop the recommendations for the guideline. Everyone had access to the original research questions, the literature that passed selection and the results of the grading process. After each member finished their part, the parts were merged together and then presented to the whole taskforce for verification and feedback.

### Consensus

Twenty-seven proposed recommendations were included in the consensus finding process via the DELPHI method [20]. This method is used to generate consensus among a group of researchers addressing potentially controversial questions [21]. In this case, all task force members received a list with the proposed recommendations. They were asked via e-voting to state if they agree or disagree and add a comment or a suggested correction. As a result, the list was sent back to the chairperson to collect all comments and evaluate the rate of agreement.

Then, the results of this first round were sent back to the task force members with the rate of agreement and the anonymized comments and suggested corrections. The members were asked a second time to state their agreement/disagreement on the 27 recommendations and add comments. After this second round, every recommendation had received a clear vote for either agreement or disagreement.

In total, 23 of the 27 proposed recommendations were accepted of which 22 reached a strong consensus. There were no recommendations supported by an intermediate consensus (70–90% agreement) and only 1 recommendation with a weak consensus (50–70% agreement). For 3 of the proposed recommendations no consensus could be achieved (< 50% agreement).

### Strength of recommendation

The strength of recommendation was determined by the task force members as either strong or weak [18].

## Scientific background for CPR in microgravity

Modern resuscitation techniques originate from the research projects of Kouwenhoven, Jude, and Knickerbocker in the 1950s and 1960s [8] and have been an active area for medical research [22, 23].

With humankind starting the exploration of space in 1961, medical emergencies during spaceflight have been a possibility, leading to space agencies actively preparing and training their crews for such events [24]. The first available reference to CPR in space dates back to 1968 when Busby published his report about medical problems for future space missions [25]. Busby suggested the application of rescue breaths or mask ventilation complemented with chest compressions for astronauts suffering acute hypoxia, but did not elaborate on his suggestion [25]. In addition to that, Frey et al. proposed an emergency room for future space stations in which chest compressions could be performed [26]. NASA took on the challenge and performed experiments with different positions to perform chest compressions in 1992 and even tested a mechanical chest compression device [27]. The Crew Medical Restraint System (CMRS) was first introduced during Space Shuttle mission STS-81 in 1997 and enabled a fairly quick and secure fixation of a patient and the provider [28].

In the following years, several experiments were conducted during parabolic flight [29–31] or in simulated microgravity [32–36] in order to identify the ideal technique of performing chest compressions.

Until today, no cardiac arrest ever happened in space that was not associated with a catastrophic accident and consecutive loss of the whole spacecraft and crew.

### Microgravity CPR methods

So far, five different techniques for CPR in space have been described and evaluated. Certainly, the restrained CPR method in the standard position represents the most apparent approach to performing chest compressions in microgravity, as it is simply the application of the earth-based standard technique. It requires the rescuer and the patient to be fastened to the CMRS. The rescuer utilizes one strap around the waist and one strap across the lower legs to keep their position at the side of the patient’s torso [37]. It was one of the first techniques to be investigated during parabolic flight [30].

In the straddling position of the restrained CPR method, patient and rescuer are again both attached to the CMRS. The difference lies in the rescuer kneeling across the patient’s waist and performing chest compressions on top of the patient. This leads to a significant reduction in the required space and could represent an advantage in a spacecraft where space is limited [37].

The reverse bear hug method (RBH) represents a modified version of the Heimlich-maneuver with the rescuer enclosing the patient’s chest from behind. This technique lends itself for CPR immediately at the site of the cardiac arrest, as it does not require the patient and rescuer to be restrained. Obviously, this technique cannot be performed with a patient restrained on the CMRS [30].

Like the RBH method, the handstand (HS) method does not require the patient to be restrained on the CMRS, although it can be performed, however, on a restrained patient. The patient is placed with their back on a solid surface of the spacecraft. The rescuer then places their feet on an adequate surface on the opposite wall, arms stretched out above the head. With both hands placed in the correct position on the patient’s sternum, the rescuer flexes and extends their hips and knees to generate the force for compressing the patient’s chest.

This technique comes with the advantage of using the rescuer’s lower extremity muscles, which enables higher exercise endurance. Nevertheless, the major disadvantage is represented by the dependence on the distance between the patient and the opposing surface as well as the actual height of the rescuer. Those factors make the HS method a very uncertain technique in an emergency [37].

The newest technique is the Evetts-Russomano (ER) CPR method. Like the HS and RBH methods, it does not require a restrained patient and is suitable as a first-aid instrument at the site of the emergency. The rescuer places their left leg over the patient’s right shoulder and the right leg around the patient’s torso. By interlocking their ankles in the center of the patient’s back, the rescuer attaches himself to the patient and can now generate force onto the patient’s chest without being pushed away [32, 37].

The technique’s advantages are quick application, independence from spatial circumstances, and easy access to the patient’s airway. However, without restraint rescuer and patient could drift and collide with the sides of the spacecraft and, thus, pose a potential risk.

Besides the microgravity CPR techniques mentioned above, other chest compression techniques, like the Mackaill-Russomano-technique, have been suggested for the special envirnonment of hypogravity [38].

## Results and recommendations

### Should CPR in microgravity be divided in different phases?

*Recommendation 1: CPR in microgravity SHOULD be divided into a chain of survival consisting of Basic Life Support (BLS) and Advanced Life Support (ALS).*

### Evidence summary

Several studies investigated the feasibility, course of action, and effectiveness of CPR in microgravity [29–36, 39, 40]. It consists of early recognition and the call for help, early and effective chest compressions, early advanced life support with the use of a defibrillator if applicable and high quality post resuscitation care [11, 41]. The chain of survival is a central part of the terrestrial CPR guidelines [11, 42].

### Rationale for the recommendation

Emergencies can happen anywhere - this includes also any place of a spacecraft. To guarantee effective and immediate CPR during spaceflight, we recommend a two-step approach similar to the earth-based CPR guidelines with a first-aid component (BLS) and a second component once additional help arrives (ALS) [11, 41]. This includes the recommendation of a chest compression technique for BLS that does not require patient or rescuer to be restrained or is dependent on the distance between two surfaces (such as the HS-method) and can therefore be initiated, immediately and without any equipment. Another advantage of this approach is that once help arrives, transport of patient and rescuer under ongoing chest compressions by the additional crew members is possible.

### What is the best initial chest compression technique at the site of the emergency?

*Recommendation 2: For initial BLS at the site of emergency, the Evetts-Russomano method (ER) SHOULD be applied initially. If the rescuer cannot perform adequate chest compressions with the ER method, the rescuer should switch to the Reverse-Bear-Hug method (RBH).*

### Evidence summary

Several controlled trials analyzed which chest compression technique is the most effective in microgravity [40, 43]. For initial BLS at the site of the emergency, only a technique that does not require patient or rescuer to be restrained can be considered. This limits the possible techniques per se to the Handstand-method, the Reverse-Bear-Hug method and the Evetts-Russomano method. Only one controlled trial compared those three methods [33], whereas other studies concentrated on one or two of the techniques [29, 30, 32, 36] or analyzed the effectiveness mathematically [40].

Concerning the compression rate, the ER technique is superior to the RBH method (compression rate, 104.6 ± 5.4 bpm vs. 94.7 ± 5.4 bpm) [40]. The HS method resulted in compression rates of more than 120 bpm (applying the 2010 ERC guidelines) [33] and in one instance of just below 100 bpm [30].

With regard to compression depth, the HS method demonstrated the highest depth (47.4 ± 2.4 mm) [33] of all the compared techniques, although it still not reached the recommended 50–60 mm of the current ERC guidelines [44]. The ER method showed inconsistent results concerning compression depth, ranging from 45.7 ± 2.4 mm [32] to 27.1 ± 7.9 mm [36]. Finally in the RBH method, compression depths with a maximum of only 41.7 ± 6.2 mm [33] were achieved.

### Rationale for the recommendation

Although the HS method proved to deliver the most effective chest compressions with regards to the 2010/2015 ERC guidelines [36, 40], we recommend the ER method as the primary technique for basic life support in microgravity. The decision is based on advantages of the ER method which clearly outweigh its disadvantages.

In contrast to the HS method, the ER method is not dependent on a specific spacecraft diameter. Another advantage of the ER method is that it allows the patient and rescuer to be transported under ongoing chest compressions by a third person. The focus lies on the transportation aspect, as in a spacecraft the emergency equipment would be generally stored near the crew medical restraint system.

Consequently, it would be more effective to transport the patient to the equipment and a restraint system thus creating the best treatment environment possible. This is the reason for the recommendation to apply the RBH method in case of a failure of the ER method.

### What is the best chest compression technique at the designated emergency treatment site?

*Recommendation 3: As soon as the patient has been restrained on the Crew Medical Restraint System chest compressions SHOULD be applied using the Handstand-method (HS) if favored by the dimensions of the spacecraft and provider height.*

### Evidence summary

As mentioned above, the HS method has proved to deliver the highest quality manual chest compressions in microgravity [34, 40]. With HS, both compression depth (44.9 ± 3.3 mm) and compression rate (115.4 ± 12.1 bpm) were superior to all the other manual chest compression techniques [40]. Furthermore, it has been demonstrated to be the least strenuous technique, with a lower minute ventilation for the rescuer (HS 39.89 ± 2.01 l/min vs ER 58.38 ± 2.90 l/min vs RBH 48.24 ± 2.32 l/min) [34].

### Rationale for the recommendation

As the HS method represents the most effective chest compression technique in microgravity [40], it should be regarded as the current gold standard. Thus, it should be applied as soon as the situation allows, once patient has been transported to the emergency equipment and restrained to the CMRS. This implies that the CMRS is installed at a site of the spacecraft where the distance from the CMRS to the opposing wall enables the crewmembers to perform chest compressions in the HS-method.

Theoretically, the HS method could be applied anywhere in the spacecraft if the surface distance allows its application. Furthermore, transportation of the patient and rescuer to the CMRS and emergency equipment under ongoing HS chest compressions is impossible. This justifies our recommendation to only use the HS method once the patient has been restrained to the CMRS.

### Which chest compression technique should be used at the designated emergency treatment site if the HS method cannot be used?

*Recommendation 4: If the application of the HS method seems impossible either the restrained CPR method using the standard OR straddling position SHOULD be applied.*

### Evidence summary

There were only two studies that investigated the use of the standard or straddling position for CPR in microgravity with the rescuer and patient being restrained [30, 31]. Both studies were performed during parabolic flight, one using a mannequin [30], the other a using a swine model [31]. Overall, the performance of chest compressions in those standard or straddling positions was inferior to the HS method (compression depth HS 4.01 ± 0.51 cm vs. standard 1.98 ± 1.12 cm vs. straddling 3.07 ± 1.19 cm) [30] However, the achieved compression rate for all mentioned techniques was equally good [30].

### Rationale for the recommendation

If limitations in space or other factors prevent the rescuer from applying chest compressions in a restrained patient using the HS method, it is necessary to apply chest compressions in either the restrained standard or restrained straddling position. Although the findings suggest an advantage of the straddling over the standard position [30, 40], we intentionally do not recommend one technique over the other.

In a situation where the HS method cannot be applied, there will possibly be other factors affecting the application of chest compressions to the patient. Those factors could include limitations in available space, wall-to-CMRS distance and human physiological limitations (e.g. height of the rescuer). In those cases, the rescuer must decide which of the two techniques can be applied and will provide the most clinically effective chest compressions [37].

#### Automated Chest Compression Devices (ACCD)

In recent years, the invention of ACCDs has brought an innovative aspect for the application of cardiopulmonary resuscitation [45, 46]. This invention was mainly driven by the fact that quality of manual chest compressions is one of the major contributors to good patient outcome in CPR [47]. However, the quality of manual CPR is influenced by many factors (height, weight, training) and deteriorates significantly within minutes even in highly trained and fit rescuers [48–50]. To overcome those limitations, ACCDs were developed by various companies to improve the delivery of consistent high-quality chest compressions over longer periods. Nevertheless, no study was able to show an advantage of ACCDs over manual chest compressions [51].

### Should an automated chest compression device be used on a patient in cardiac arrest in microgravity?

*Recommendation 5: An automated chest compression device COULD be used on a restrained patient (if available). Its installation, however, should not delay high quality chest compressions.*

### Evidence summary

Several studies investigated the safety and effectiveness of ACCDs in earth-based clinical and preclinical use during CPR [52, 53]. Unfortunately, no ACCD has ever been tested under actual microgravity conditions during spaceflight. Only one abstract reports the testing of a LUCAS-II ACCD during parabolic flight, although no full-length publication could be retrieved [54]. In this study, the ACCD was already fastened to CPR manikin when microgravity was reached. There was no significant difference in compression depth and rate found while comparing microgravity with 1G conditions. This indicates that an ACCD could potentially operate effectively in microgravity.

### Rationale for the recommendation

The authors recognize the potential benefits of ACCDs in microgravity with special consideration of the challenging application of manual chest compressions. Fatigue, suboptimal spatial conditions and limitations in the number of rescuers can limit the effectiveness of manual chest compressions even under optimal conditions in microgravity. Therefore, ACCDs represent a potential solution to overcome those human limitations.

The reason behind the weak recommendation is based on the lack of high-quality studies for the use of those devices in microgravity. Another factor is vibrations being caused by those devices whose effects on the structural integrity of a spacecraft are challenging to extrapolate without further investigations. Moreover, the crew training to apply these devices must be extensive and the process of fastening the patient to such a device is likely to be time-consuming, especially in microgravity. Furthermore, the weight and size of ACCDs represent a likely limitation for the inclusion of those devices to the medical kit.

Although cardiac arrest remains a possibility during spaceflight, its estimated occurrence is very low. This argues against embarking the load of > 8 kg during a space mission for an ACCD that might never be used. In doing so, other medical or mission-relevant equipment might be left behind. This question cannot finally be answered by this research group but will need to be addressed for future space exploration missions.

#### Airway management and ventilation

Another crucial part in the delivery of successful CPR is ventilation [55, 56]. Besides effective chest compressions and defibrillation, it constitutes the central skill of Basic and Advanced Cardiac Life Support [23]. Therefore, it seems reasonable to encompass the topic of airway management and ventilation in these guidelines.

As future space missions might not include trained anesthesiologists, nurse anesthetists, emergency physicians, or paramedics, who have sufficient skills in airway management during emergencies, a reasonable strategy for CMOs and regular crewmembers is needed.

### Which airway device should be used to ventilate a patient during CPR in microgravity?

*Recommendation 6: If no rescuer with extensive training in tracheal intubation is present, a second generation supraglottic airway device SHOULD be used for airway management.*

### Evidence summary

We have identified three studies investigating airway management in microgravity [57–60]. Of those three studies, two are covering supraglottic airway devices as well [59, 60]. In particular, the laryngeal mask (LMA), as a representative of the supraglottic airway devices (SGA/SAD), showed a high successful insertion rate in simulated microgravity of 100% in a restraint position and 97.5% in a free-floating position [60].

Although the study subjects of Keller et al. were trained anesthesiologists, their success rate for tracheal tube (TT) intubation in a free-floating condition was low in some situations (15%). Even under restrained conditions several intubation attempts were needed (successful insertion rate: 92.5%) [60].

### Rationale for the recommendation

SGAs are an accepted and widely used airway device during CPR [42]. Although the CPR guidelines of the ERC recognize the tracheal intubation as the optimal method for airway management, they acknowledge the difficulty and dangers of ETT intubation by inexperienced providers. Therefore, SGAs represent a valid and safe alternative that can be effectively applied by inexperienced providers with minimal training under CPR conditions [61].

This recommendation was made intentionally although no study investigated a second generation SGA. However, we believe that the differences in handling and effectiveness of first- and second-generation SGAs are minimal and the experiences with the first generation devices are sufficient to make this recommendation.

### Which airway device should be used if a provider experienced in tracheal intubation is present?

*Recommendation 7: The tracheal intubation remains the gold standard for securing the airway if performed by a skilled provider and SHOULD be performed in that case.*

### Evidence summary

In total, three studies investigated the feasibility and success rate of TT intubation in microgravity [58–60]. Another study was published during the process of guideline development [62, 63]. Although all studies were performed with limited subject numbers, it could be shown that TT intubation in microgravity is possible, but the success rates differed in the studies.

Starck et al. is the first study to investigate the use of a video laryngoscope [62]. They measured an 80% success rate for oro-tracheal intubation by novice operators with a video laryngoscope in microgravity, compared to a 40% for conventional laryngoscopy. Experts performed similarly well with conventional and video laryngoscopes.

Three older studies investigated conventional laryngoscopes. Whereas Keller et al. reported of an TT intubation success rate of 92.5% under constraint conditions in simulated microgravity [60], Groemer et al. only found a 33% success rate for TT intubation under constraint conditions [58]. This difference could be explained by comparing the study designs: Groemer et al. researched TT intubation during parabolic flight - with a limited microgravity time of 23 s [58], whether Keller et al. performed TT intubation in a submerged scenario with no time limit in simulated microgravity and an average time to successful intubation of 36 s [60]. Rabitsch et al. found a 86% success rate for TT intubation in a restraint setting under microgravity conditions by paramedics [59].

### Rationale for the recommendation

Tracheal intubation represents the gold standard in the international guidelines concerning airway management during cardiopulmonary resuscitation [42]. However, crew-training to guarantee high success rates for TT intubation needs to be extensive and requires at least 50 tracheal intubations in humans to reach a 90% success rate [64]. Therefore, if a crewmember possesses the necessary training and experience, he or she should attempt to perform TT intubation in a patient in cardiac arrest.

However, the authors realize that such an instance will be unlikely, and SGAs represent a viable alternative in lack of a crewmember trained in TT intubation and the required equipment.

### Should patient and rescuer during an intubation attempt be restrained or free-floating?

*Recommendation 8: When tracheal intubation is attempted patient and rescuer should be restrained using the Crew Medical Restraint System.*

### Evidence summary

Of the four conducted studies, two compared the application of TT intubation under free-floating and restrained conditions in microgravity [57, 58, 60]. Keller et al. showed a clear superiority of the restrained method (88% success rate) compared to the free-floating method (15% success rate) [60]. Groemer et al. reported about a slightly reduced success rate for the restrained method (33%) compared to the free-floating method (41%). Starck et al. only investigated TT intubation in a setup with both patient and rescuer being restrained, but showed a high success rate for video laryngoscopy in novices and experts (80% vs. 95%) [62]. Also experts achieved a high success rate with direct laryngoscopy (96%), whereas novices had a significantly lower success rate with direct laryngoscopy (40%) [62].

### Rationale for the recommendation

Given the high success rate for TT intubation under restrained conditions in the studies of Starck [62] and Keller [60] et al. and the low success rate in free-floating conditions, it seems reasonable to recommend the restrained position to perform ETT intubation in microgravity.

### Should a suction device be available during airway management?

*Recommendation 9: A manual suction device SHOULD be included in the emergency kit and be readily available during CPR, especially during airway management.*

### Evidence summary

Thus far, no study has investigated the use of a suction device during airway management in microgravity. However, several studies investigated surgical procedures and bleeding control in microgravity [65–68]. In some of those studies, suction devices were successfully used.

McCuaig et al. investigated the feasibility of blood removal in microgravity during parabolic flight and effectively used a commercial Laerdal® suction device [66]. In a different study, McCuaig et al. tested an experimental suction machine incorporating a centrifugal mechanism successfully during microgravity in parabolic flight [67].

### Rationale for the recommendation

The ability to remove fluids from a patient during airway management represents a crucial skill in emergency medicine [42, 69]. Although no studies have been performed to test suction devices during airway management in microgravity, the effectiveness of suction devices in other experimental settings during microgravity has been tested. We, therefore, recommend keeping a microgravity compatible suction device readily available during emergency airway management in microgravity.

#### Defibrillation

##### When should a defibrillator be used?

*Recommendation 10: A defibrillator SHOULD only be used on a patient that is restrained to an electrically isolated and safe surface.*

##### Evidence summary

No study specifically investigated the use of a defibrillator on a patient in cardiac arrest in microgravity. However, a defibrillator has been included in one study that concentrated primarily on the provision of chest compression [31]. In this study, a commercially available defibrillator (Physio-Control, Lifepack 10, Redmond, WA) was reliably used in the swine model. The subjects were fastened on the electrically isolated surface of the CMRS [31].

##### Rationale for the recommendation

Defibrillation represents a crucial step in the treatment of a patient in cardiac arrest suffering from ventricular fibrillation (VF) or pulseless ventricular tachycardia (pVT) [42, 70, 71]. A defibrillator was first introduced on a Space Shuttle mission with STS-90 in 1998 [31] and is part of the crew health care system (CHeCS) on the ISS [28]. Although defibrillation offers great advantages in patient care and is a potentially a lifesaving procedure, it also represents a hazard for the rescuer and other crewmembers [72–74]. Therefore, it is necessary to reduce the risk of an accidental shock for the other crewmembers and potential damage to the electrical systems of the spacecraft. For this reason, we recommend the use of a defibrillator only on a patient that has been restrained on an electrical isolated surface such as the CMRS [28].

##### What kind of defibrillator and electrodes should be used on a patient in cardiac arrest in microgravity?

*Recommendation 11: An automated external defibrillator (AED), with long duration batteries and long shelf-life self-adhesive pads, SHOULD be stored with the emergency equipment.*

##### Evidence summary

No study has been published that has investigated the use of an AED for CPR in microgravity. However, as already mentioned above a defibrillator has been reliably tested during parabolic flight [31].

##### Rationale for the recommendation

A defibrillator is an integral part of the emergency equipment during spaceflight. Therefore, it should be stored with the rest of the emergency equipment to guarantee quick deployment of this device. We recommend the use of self-adhesive defibrillation pads as they offer the advantage of better handling especially in microgravity since they require no contact pressure (vs. conventional paddles) so the rescuer can keep their hands free for other tasks.

##### What features should the AED contain?

*Recommendation 12: The AED SHOULD have a user-friendly interface, a step-by-step instruction voice for correct pads positioning and electrical shock delivery and a timing device for correct chest compressions/ventilation rate.*

##### Evidence summary

As already mentioned above, evidence for defibrillator use in microgravity is scarce. However, a study has been conducted investigating the use and effectiveness of a timing device for CPR by ISS astronaut CMO analogues [39]. Although this study was performed in normogravity, it showed that CMOs with only a short medical training would highly profit in their performance of CPR through the application of a timing device like a metronome for chest compressions and ventilation [39].

##### Rationale for the recommendation

As it is not mandatory for the crew of a spacecraft to include a health care provider skilled in emergency medicine, the current standard for medical care during space missions is represented by the CMO [28]. Those CMOs receive a minimum of 80 h medical training with only a small part being focused on CPR. Therefore, it seems reasonable to include an AED in the medical kit of a spacecraft. The AED guides the CMO (or other crewmembers) with visual and acoustic instructions through the process of CPR.

##### How should crewmembers be trained for AED use?

*Recommendation 13: All crewmembers SHOULD be trained in the use of the specific AED provided during the mission.*

##### Evidence summary

To this day, no study has been performed to investigate training issues for defibrillation during spaceflight.

##### Rationale for the recommendation

There are different models of AEDs available on the market. It seems only logical to include the very same AED model in the preflight training that is issued on the actual mission. For laypersons short training sessions of around half an hour with AEDs have been showing good results for long-term skill retention [75].

##### Should CPR be attempted in the absence of a defibrillator?

*Recommendation 14: Even if survival is highly unlikely without defibrillation, CPR SHOULD start when a defibrillator is unavailable in the space vehicle, in patients who appear to be in cardiac arrest.*

##### Evidence summary

Studies concerning defibrillator use in microgravity in particular are almost not available. As no cardiac arrest ever happened during spaceflight, no outcome investigations are possible.

##### Rationale for the recommendation

There might be different situations in which the on-board defibrillator might not be available, e.g. a technical defect. Even in the absence of a defibrillator the other crewmembers should nevertheless perform CPR with the available equipment. On the one hand, the cardiac arrest could potentially be caused by an asystole or a pulseless electric activity (PEA) and consequently will not be eligible for defibrillation therapy.

On the other hand, a different potentially reversible cause for the cardiac arrest could be present. After successful therapy of this reversible cause it could be possible to establish a Return of Spontaneous Circulation (ROSC) even without a defibrillator.

##### Should defibrillation be attempted, even if survival is unlikely?

*Recommendation 15: Although the survival rate is likely to be reduced in the absence of medical skills and/or equipment for on-going medical support in the event of ROSC in microgravity, defibrillation SHOULD take place when appropriate.*

##### Evidence summary

So far, most studies on CPR in microgravity have been designed towards the actual skill performance. No study investigated adjunct measures or post resuscitation care during spaceflight. One study concentrated on a statistical approach to estimate outcomes of astronauts with myocardial infarction and subsequent cardiac arrest during spaceflight [76]. The authors estimate the survival rate of an astronaut suffering from sudden cardiac arrest to be lower than 5% [76].

##### Rationale for the recommendation

The context of a space mission represents a highly remote setting. The crew might comprise only of members with limited medical training and medical equipment and medications are scarce with no option for resupply. Furthermore, external help and evacuation is unavailable or difficult. These factors impair the prognosis of a patient in ROSC as no further treatment in an advanced medical facility is available.

As the cause of the arrest is sometimes not obvious in the emergency treatment, we recommend delivering full ALS therapy for a patient in cardiac arrest in microgravity whenever possible, acknowledging that post resuscitation care will be suboptimal.

#### Intravenous access/ drug administration

##### When should venous access be attempted?

*Recommendation 16: Venous access SHOULD ONLY be performed if more than two rescuers are present during a cardiac arrest and high-quality CPR is performed.*

##### Evidence summary

Intravenous access in microgravity has never been investigated specifically, but it was included in different studies as a consequence of the primary intervention [65, 67]. It was shown that intravenous access could be successfully obtained [67].

##### Rationale for the recommendation

Venous access and drug therapy are integral parts of the ACLS algorithm [42]. However, these steps are less critical then chest compressions, defibrillation and ventilation. We, therefore, recommend only attempting venous access once effective CPR is ongoing.

##### Which drug administration route should be attempted first?

*Recommendation 17: As a first choice for the application of medication a peripheral venous cannulation SHOULD be used.*

##### Evidence summary

Intravenous access in microgravity has never been investigated specifically, but it was included in different studies as a byproduct [65, 67].

##### Rationale for the recommendation

The current CPR guidelines incorporate the recommendation to attempt intravenous access as a first route for drug administration [42]. As it has been proven, that intravenous access in microgravity can be established we recommend choosing it as a first route for drug administration in microgravity.

##### Which alternative should be used for venous access?

*Recommendation 18: When a peripheral venous access cannot be established in a patient in cardiac arrest in microgravity, the intraosseous tibial route SHOULD be used.*

##### Evidence summary

There has been no study conducted which investigates the performance of intraosseous access during cardiac arrest in microgravity.

##### Rationale for the recommendation

The current CPR guidelines incorporate the recommendation to establish an intraosseous access in a patient in cardiac arrest when the intravenous cannulation fails. The intraosseous route achieved similar plasma drug concentrations as the intravenous route in a comparable time [77]. Also, the training to learn and retain skills for intraosseous access is moderate [78] and equipment for its application is readily available. We, therefore, recommend the application of intraosseous access as a second line for failed intravenous access attempt on a patient in cardiac arrest in microgravity, although it has never been tested in microgravity.

##### How should an infusion be administered?

*Recommendation 19: For intravenous and intraosseous infusion, a degassed infusion bag encased in a pressure bag SHOULD be used.*

##### Evidence summary

No study specifically investigated the best way to administer intravenous fluids in a patient in cardiac arrest. However, they were tested in an ATLS study in microgravity during parabolic flights [65]. In this study, degassed infusion bags encased in a pressure bag were effectively used to administer crystalloid fluids [65]. In a different study, an electric infusion pump was successfully used to administer intravenous fluids in a mannequin model [67].

##### Rationale for the recommendation

Different options are available for continuous infusion of intravenous fluids. One is utilization of an infusion pump to administer intravenous fluids. Another option would be the degassed infusion bag encased in a pressure bag. The advantage of the pressure bag lies in its simplicity. As it does not rely on electrical power and an electrical motor, it offers fewer failure possibilities. Also, the application time of a simple pressure bag is probably shorter than the time to prepare and start an advanced infusion pump. We therefore recommend the use of a degassed infusion bag encased in a pressure bag for quick administration of intravenous fluids in the emergency situation of a cardiac arrest in microgravity.

#### Telemedicine

##### Should telemedicine support be consulted in LEO?

*Recommendation 20: In low earth orbit telemedicine support SHOULD be consulted in the event of a cardiac arrest, when it seems feasible and the manpower for its application is present.*

##### Evidence summary

No study directly investigated the use of telemedicine support during CPR in a patient in cardiac arrest in microgravity. However, telemedicine support in the performance of emergency medical procedures such as chest drain insertion has been successfully tested [65].

##### Rationale for the recommendation

In the terrestrial setting, telemedicine is an accepted addition for emergency therapy in critically ill patients [79, 80]. However, CPR requires immediate action of all available crewmembers and emphasis should be put on the onboard best quality treatment of the patient. In the first minutes of a cardiac arrest the crew will be fully engaged to perform the CPR algorithm and the skills necessary to do so.

Also, telemedicine support will probably not be able to positively influence the application of these crucial first CPR steps. Therefore, we believe, that the focus should lie on the autonomous application of CPR by the crewmembers. Only if CPR can be performed without endangering quality, one member of the crew should try and seek telemedicine support.

##### Should telemedicine support be consulted during space exploration missions?

*Recommendation 21: During space exploration missions to Mars telemedicine support will be impractical during CPR due to the communicational time-delay (3–23 min) and SHOULD only be attempted, when additional crewmembers, not involved in treating the patient, are present.*

##### Evidence summary

Communication delay from Mars to Earth is estimated to range between 3 and 23 min each way [81–83].

##### Rationale for the recommendation

Because of the long time delay no relevant benefit from telemedicine support in the initial phase of CPR is expected. A response from Mission Control would subsequently take 6 to 46 min. As successful resuscitation attempts often last less than 15 min until ROSC [84, 85], it is highly unlikely that telemedicine support will have a beneficial impact in the initial phase of CPR treatment. However, the crew will be obliged to inform Mission Control of an ongoing medical emergency.

##### Who should make the decision to terminate CPR if necessary?

*Recommendation 22: The decision to terminate resuscitation SHOULD be made by the crewmember with the highest medical qualification after consultation with telemedicine support. Only if telemedicine support is unavailable or time delay prevents prompt feedback the decision has to be made by the crewmember with the highest medical qualification alone.*

##### Evidence summary

There are no studies investigating the termination of CPR in a patient in cardiac arrest during spaceflight. In addition, this topic remains highly controversial even in the terrestrial setting.

##### Rationale for the recommendation

The topic of termination of CPR remains a highly controversial one, even in the terrestrial setting [86–88]. Especially for a crew in spaceflight, the decision to terminate a CPR attempt will be extraordinary challenging because of the close personal relation to the patient and the remote setting. As a physician might not be part of the crew, either another crew member with special medical training (nurse, paramedic, etc.) or a CMO will oversee resuscitation. It is subsequently their duty to terminate the treatment if the situation is deemed futile.

This decision surely requires communication with the crewmembers involved in the treatment. Furthermore, it should be attempted to consult the telemedicine support on this decision. If telemedicine support is unavailable or communication delay appears to be inadequate to the medical leader, the decision to terminate CPR should be made, nonetheless. It is their obligation to the crew to prevent ongoing CPR in a futile situation and to preserve the medical equipment and drugs for future possible emergencies.

#### Ethics

Next to recommendations concerning the best clinical practice for successful CPR during spaceflight, ethical and psychological / psychiatric issues deserve attention in this context; particularly since peculiarities of CPR are entangled with specific characteristics of space flight. While there is some evidence regarding psychological, neuro-behavioural aspects [89] and ethical issues of space flight [90], there is no guidance available to date about specific considerations in the context of CPR. The issues raised in this section are therefore not intended as recommendations, but rather as questions for reflection, and as future research directions.

#### Ethical questions related to CPR

Futility is a major ethical issue discussed in the context of CPR on Earth – i.e. if an intervention is considered to be futile in terms of its benefit for length and quality of life, its ethical justification is questionable. However, there are no clear boundaries for futility; this may even be aggravated in the context of space flight since the death of one crew member may jeopardise the whole mission, resulting in potential conflicts of interest between the crew and the person at risk of death. Moreover, decision-making is complicated by the fact that it will be virtually impossible to involve proxies (e.g. family members) due to remote communication with the ground.

The AHA recommends considering the values of patients, healthcare providers and local society in determining the appropriateness of CPR for an individual patient [91]. Given the particular normative, cultural, and spatial conditions of space flight, a context-specific ethical framework is needed to support ethically responsible decision-making concerning the application and continuation of CPR during space flight. This requires discussing acceptable and sustainable values regarding CPR, including questions of futility, dignity, information of the family, and proactive strategies such as advance directives / “Do Not Attempt CPR” (DNACPR) orders. An ethical framework also involves strategies to anticipate or assess the presumed will of the person in need of CPR, as well as measures to be taken for crew members in order to be prepared for the challenges related to such a decision, and to cope with the decision and its outcomes both as individuals and as a ‘community of destiny’. In addition, pre-mission training should include reflection on diverse scenarios (e.g., survival of the crew member, survival with impairment, or death).

Another ethical question regards the adequate and respectful dealing with the newly dead in case of unsuccessful CPR. This includes the challenge of informed consent with the use of newly dead patients for research and training, and the balancing of interests between the protection of medical confidentiality and the interest in gaining better insight in medical issues in space through disclosure of information and systematic collection of data [90].

#### Perceptual, cognitive, and psychomotor performance

For CPR on Earth, as is true for every emergency situation, a number of highly specific conditions, skills, and psychophysiological prerequisites are essential. Next to semantic and episodic knowledge on how to perform CPR, its application demands (a) sympathetic function such as attention, focused concentration, and quick response capacity; (b) effective motor function, in particular fine motor skills; and (c) cognitive function in terms of interpersonal interaction and decision-making. In the remote microgravity environment, several factors are complicating psychophysiological, cognitive, and motor function.

For example, it was reported that the effects of microgravity in addition to the working and living conditions of space can induce stress states in astronauts that also can lead to degradations of cognitive and psychomotor performance [92]. Research on earth has shown that the perceptual motor performance deteriorates under stress [93–95], particularly with regard to control processes such as the perception of time [96, 97], the relationship between automatic and controlled processes (inhibitory processes), and the categorization of incoming information [98, 99]. A similar decrease of the perceptual motor performances in the stressful environment of a space mission could be confirmed [100–109], but it remains unclear to what extend these observed deficits will change during long- term space missions. Moreover, findings suggest that increased feelings of loneliness and abandonment may interfere with the cognitive performance [110] and that executive functions [111–114] and decision making [115] are impaired in space missions.

Furthermore, the effect of microgravity is related to mechanical and proprioceptive changes during the execution of movements, leading to a disruption of the usual relationships among efferent and afferent signals that has been referred to as a state of “sensorimotor discordance” [103]. With regards to CPR, the question could be stressed whether these limitations also significantly affect cognitive and psychomotor skills during resuscitation. However, the question also arises as to how significant these limitations are and that, of course, resuscitation under the above-mentioned limitations is better than no resuscitation at all. Considering situational restrictions concerning decision-making, structured guidelines for emergency situations like CPR are urgently needed.

#### Intercultural issues

As a specific aspect of group dynamics, the international and intercultural nature of space flight needs to be considered. Crews are diverse in terms of ethical and cultural norms, e.g. regarding the broad ethical principles of beneficence, non-malevolence, autonomy, and justice. For example, there may differences in emphasis on patient autonomy or informed decision-making. There may also be culturally diverse values and beliefs concerning end-of-life, dealing with deceased persons, handling emotions, spirituality, rituals of bereavement, social behaviour norms [116]. More specifically with respect to CPR, this may have implications for questions concerning the beginning and ending of CPR [91]. Moreover, there may be different manifestations of emotional reactions to distressing situations [116] which may pose a challenge in terms of interpersonal communication and group dynamics.

#### Possible prevention and treatment approaches

During long-duration expeditions, where higher crew autonomy and communication delays with the Earth will be unavoidable, the ability to deal with psychiatric problems on board becomes especially important. There will be no possibility to evacuate an ill crewmember and the communication delays will make real-time conversation with specialists back home impossible. As a consequence, support will depend on crew members being trained in psychological counselling and the use of psychoactive medications. This person should have a knowledge of [1]: individual psychopathology and small group behaviour [2]; the individual and interpersonal effects of stressors to be expected during the mission [3]; crisis intervention techniques and the facilitation of group awareness, cohesion, and teambuilding; and [4] the appropriate use of tranquilizers and other psychoactive medications, including their usefulness and side effects under conditions of microgravity [117, 118]. Studies suggested that computer-based intervention programs applying cognitive-behavioural and self-help instruction may be as effective as face-to-face intervention for dealing with mild types of psychopathology [119–121]. Pre-mission trainings should be implemented by each space agency based on international agreements and focus on further development of coping abilities of individual crewmembers and crews [89].

According to evidence from space and other isolated and confined settings, intra crew tension, leadership styles, and group dynamics are key factors responsible for exacerbating or ameliorating stress, or facilitating coping and adaptation [118, 122] and should be included in these training programs, as well as self-care and self-management and cross-cultural aspects in the preparation for long-term missions.

#### Conclusion and future research directions

We argue that for an adequate consideration of ethical and psychological issues surrounding CPR during space flight, a multi-level approach is needed. An important systemic prerequisite will be a general ethical and psychological framework that allows for a culture of constructively dealing with fragility and finiteness in a context where fitness, strength, and controllability are key requisites. This already starts with the mission selection process, when fear of disqualification may result in underreporting of medical problems [90]. Pre-mission trainings should address ethical issues and psychological coping strategies in emergency situations. Moreover, context-specific prevention and treatment approaches need to be ensured, as well as the presence of crew members trained in counselling and the use of psychoactive medication. Future research should address these ethical and psychological tension fields in order to elucidate expedient starting points for intervention. Herein, participatory approaches involving key stakeholders may be fruitful to initiate and maintain dialogue with respect to the conditions that are needed to create an ethically and psychologically sustainable environment of care in the case of CPR.

## Summary and conclusions

This guideline is intended to summarize the available evidence for the application of CPR in a patient in cardiac arrest in microgravity. It is an early attempt at improving CPR performance in this special setting. The authors acknowledge that a cardiac arrest during spaceflight is a highly unlikely scenario at the moment. As it has never happened, real life experience is missing. But, with the future rise of space tourism and the increase of missions to the Moon and potentially to Mars, cardiac arrest onboard a spacecraft is a real possibility.

Several studies have assessed CPR techniques in microgravity. However, most of those studies concentrated on the application of chest compressions. In contrast, there are only four studies investigating airway management in microgravity. The evidence for many other aspects of CPR such as defibrillation, drug therapy or post resuscitation care remains minimal to non-existent. Therefore, this guideline faces also some important limitations.

Furthermore, the available studies were either performed in microgravity during parabolic flights, during simulated microgravity in a body suspension device or underwater. All of those settings pose their own limitations. Parabolic flight only allows for 20–22 s of microgravity and, thus, many experiments are unable to investigate the application chest compressions over realistic periods of time (2-min duration of a standard chest compression cycle).

Body suspension devices allow the observation during longer periods, but only simulate an imperfect microgravity since the subjects are still affected by Earth’s gravity. Underwater scenarios are also used to simulate microgravity. If neutral buoyancy in the experimental setup is achieved, microgravity can be simulated. But the necessary preparations concerning equipment (buoyancy control device, regulator, diving goggles, etc.) and handling limit the transferability of such a scenario to actual spaceflight.

CPR in microgravity is feasible. However, it requires modifications of the terrestrial guidelines to consider the unique challenges of microgravity and long duration spaceflight. Not only does the lack of gravity dictate special techniques for chest compressions, the clinical resources are extremely limited. This is primarily caused by the unavailability of imminent support and evacuation capabilities. Furthermore, limitations concerning equipment and training restrict the availability of proper post resuscitation care in form of an intensive care unit. Therefore, and in the absence of any scientific evidence for intensive care in microgravity, no explicit recommendations concerning post-resuscitation care were made in this guideline.

One additional factor is that the main task of the crew is the mission, and medical training and preparations only make up a small part of astronaut training. They therefore have limited opportunity to develop clinical confidence required for successful CPR. A future crew for space exploration missions might not include a physician experienced in emergency medicine, or the physician might become incapacitated, which further decreases the medical capabilities of the whole crew. This guideline represents a first step to lay an evidence-based foundation for CPR in microgravity during spaceflight.

Further studies are necessary, but this guideline forms a framework for future research priority setting in emergency space medicine. However, the upcoming Moon missions and the medical experiments that will be performed during those missions will hopefully enrich our knowledge on emergency medicine in microgravity. Although no one would ever hope for real-life medical emergencies, it is only a matter of time until the first cardiac arrest during spaceflight happens. It can only be our task to equip our crews with the best techniques and treatment strategies.

## Supplementary information

**Additional file 1.** PICO-questions, literature search strings for PUBMED and retrieved hits as of 31^st^ July 2017.

## Data Availability

Not applicable

## Abbreviations

ACCD: Automated Chest Compression Devices
AED: Automated external defibrillator
AHA: American Heart Association
ALS: Advanced life support
BLS: Basic life support
CHeCS: Crew health care system
CMO: Crew Medical Officer
CMRS: Crew medical restraint system
CPR: Cardiopulmonary resuscitation
DGLRM: Deutsche Gesellschaft für Luft- und Raumfahrtmedizin, German Society of Aviation and Space Medicine
ER: Evetts-Russomano
ERC: European Resuscitation Council
ESAM: European Society of Aerospace Medicine
ESAM-SMG: Space Medicine Group, European Society of Aerospace Medicine
GRADE: The Grading of Recommendations Assessment, Development and Evaluation
HS: Handstand
ILCOR: International Liaison Committee on Resuscitation
LEO: Low earth orbit
LMA: Laryngeal mask
NASA: National Aeronautics and Space Administration
PEA: Pulseless electric activity
PICO: Population, Intervention, Comparison, Outcome
pVT: Pulseless ventricular tachycardia
RBH: Reverse bear hug
ROSC: Return of spontaneous circulation
SAD: Supraglottic airway device
SGA: Supraglottic airway
TI: Tracheal intubation
TT: Tracheal tube
VF: Ventricular fibrillation
