# Supplementary material for: Cardiopulmonary resuscitation (CPR) during spaceflight - a guideline for CPR in microgravity from the German Society of Aerospace Medicine (DGLRM) and the European Society of Aerospace Medicine Space Medicine Group (ESAM-SMG)
Source: Scand J Trauma Resusc Emerg Med. 2020 Nov 2;28:108. doi: 10.1186/s13049-020-00793-y (PMC7607644; doi:10.1186/s13049-020-00793-y)
Supplement: Supplementary file 1 — Additional file 1. PICO-questions, literature search strings for PUBMED and retrieved hits as of 31st July 2017. [file 13049_2020_793_MOESM1_ESM.docx]

Additional file 1 PICO-questions, literature search strings for PUBMED and retrieved hits as of 31^st^ July 2017

| Number | Question | Search string | hits |
| --- | --- | --- | --- |
| 1 | Should future astronauts be informed straightforward about medical capabilities onboard (limited ICU capabilities, prognosis of CPR in space)? | ((((((("cardiopulmonary resuscitation"[MeSH Terms] OR ("cardiopulmonary"[All Fields] AND "resuscitation"[All Fields]) OR "cardiopulmonary resuscitation"[All Fields] OR "cpr"[All Fields]) OR "cardiopulmonary resuscitation"[MeSH Terms]) OR "cardiopulmonary resuscitation"[MeSH Terms]) OR ("cardiopulmonary resuscitation"[MeSH Terms] OR ("cardiopulmonary"[All Fields] AND "resuscitation"[All Fields]) OR "cardiopulmonary resuscitation"[All Fields])) OR "cardiopulmonary resuscitation"[MeSH Terms]) OR ("cardiopulmonary resuscitation"[MeSH Terms] OR ("cardiopulmonary"[All Fields] AND "resuscitation"[All Fields]) OR "cardiopulmonary resuscitation"[All Fields] OR ("cardio"[All Fields] AND "pulmonary"[All Fields] AND "resuscitation"[All Fields]) OR "cardio pulmonary resuscitation"[All Fields])) AND ((("prognosis"[MeSH Terms] OR "prognosis"[All Fields]) OR "prognosis"[MeSH Terms]) OR (medical[All Fields] AND capabilities[All Fields]))) AND ((((((("space flight"[MeSH Terms] OR ("space flight"[MeSH Terms] OR ("space"[All Fields] AND "flight"[All Fields]) OR "space flight"[All Fields])) OR "weightlessness"[MeSH Terms]) OR ("weightlessness"[MeSH Terms] OR "weightlessness"[All Fields] OR "microgravity"[All Fields])) OR "spacecraft"[MeSH Terms]) OR ("spacecraft"[MeSH Terms] OR "spacecraft"[All Fields])) OR "aerospace medicine"[MeSH Terms]) OR ("aerospace medicine"[MeSH Terms] OR ("aerospace"[All Fields] AND "medicine"[All Fields]) OR "aerospace medicine"[All Fields])) | 6 |
| 2 | Should future astronauts should make a “patient decree” before the mission with regards to the special medical circumstances of space flight (e.g. DNR, prolonged [unconsciousness](https://www.google.de/search?client=firefox-b-ab&biw=1280&bih=611&q=unconsciousness&spell=1&sa=X&ved=0ahUKEwigrO-24e_UAhULmrQKHRBeD5IQvwUIHygA) after CPR, what to do with their corpse) | (((("patients"[MeSH Terms] OR "patients"[All Fields] OR "patient"[All Fields]) AND decree[All Fields]) OR "living wills"[MeSH Terms]) OR ("living wills"[MeSH Terms] OR ("living"[All Fields] AND "wills"[All Fields]) OR "living wills"[All Fields] OR ("living"[All Fields] AND "will"[All Fields]) OR "living will"[All Fields])) AND ((((((("space flight"[MeSH Terms] OR ("space flight"[MeSH Terms] OR ("space"[All Fields] AND "flight"[All Fields]) OR "space flight"[All Fields])) OR "weightlessness"[MeSH Terms]) OR ("weightlessness"[MeSH Terms] OR "weightlessness"[All Fields] OR "microgravity"[All Fields])) OR "spacecraft"[MeSH Terms]) OR ("spacecraft"[MeSH Terms] OR "spacecraft"[All Fields])) OR "aerospace medicine"[MeSH Terms]) OR ("aerospace medicine"[MeSH Terms] OR ("aerospace"[All Fields] AND "medicine"[All Fields]) OR "aerospace medicine"[All Fields])) | 91 |
| 3 | What safety protocols onboard a spacecraft could restrict our procedures for CPR? | ((((safety) OR equipment safety[MeSH Terms])) AND ((((((((spacecraft) OR spacecraft[MeSH Terms]) OR microgravity) OR microgravity[MeSH Terms]) OR weightlessness) OR weightlessness[MeSH Terms]) OR spaceflight) OR spaceflight[MeSH Terms])) AND ((((((resuscitation) OR cardio pulmonary resuscitation[MeSH Terms]) OR cardiopulmonary resuscitation[MeSH Terms]) OR endotracheal intubation[MeSH Terms]) OR intubation) OR chest compression) | 3 |
| 4 | Could the vibrations caused by chest compressions (HS method, automated chest compression devices) endanger the structural integrity of the spacecraft? (Are there comparable scientific findings, e.g. from the use of physical training devices) | ((((((((chest compressions) OR cardio pulmonary resuscitation[MeSH Terms]) OR cardiopulmonary resuscitation[MeSH Terms]) OR resuscitation) OR cpr[MeSH Terms]) OR cpr))) AND (((vibration[MeSH Terms]) OR vibrations[MeSH Terms]) OR vibration) | 71 |
| 5 | Should CPR in space be divided into different time sections, analogous to the terrestrial guidelines (BLS🡪ALS, chain-of-survival in space)? Should the ER- (or RBH-) be mainly seen as an instrument of first aid, until the patient can be transported to and fixed on the restraint system | ((((((((((phase[MeSH Terms]) OR phase) OR stage[MeSH Terms]) OR stage) OR basic cardiac life support[MeSH Terms]) OR basic life support) OR advanced cardiac life support[MeSH Terms]) OR advanced life support)) AND (((((((((cpr[MeSH Terms]) OR cpr) OR cardio pulmonary resuscitation[MeSH Terms]) OR cardio pulmonary resuscitation) OR cardiopulmonary resuscitation[MeSH Terms]) OR cardiopulmonary resuscitation) OR resuscitation[MeSH Terms]) OR resuscitation) OR chest compression)) AND ((((((((space flight[MeSH Terms]) OR space flight) OR microgravity[MeSH Terms]) OR microgravity) OR spacecraft[MeSH Terms]) OR spacecraft) OR aerospace medicine[MeSH Terms]) OR aerospace medicine) | 40 |
| 6 | Should the HS-technique be mainly seen as an instrument of advanced CPR for a patient strapped to the restraint system? | (((("restraint, physical"[MeSH Terms] OR restraint[All Fields]) OR "immobilization"[MeSH Terms]) OR ("immobilisation"[All Fields] OR "immobilization"[MeSH Terms] OR "immobilization"[All Fields])) AND (((((((("cardiopulmonary resuscitation"[MeSH Terms] OR ("cardiopulmonary resuscitation"[MeSH Terms] OR ("cardiopulmonary"[All Fields] AND "resuscitation"[All Fields]) OR "cardiopulmonary resuscitation"[All Fields] OR "cpr"[All Fields])) OR "cardiopulmonary resuscitation"[MeSH Terms]) OR ("cardiopulmonary resuscitation"[MeSH Terms] OR ("cardiopulmonary"[All Fields] AND "resuscitation"[All Fields]) OR "cardiopulmonary resuscitation"[All Fields] OR ("cardio"[All Fields] AND "pulmonary"[All Fields] AND "resuscitation"[All Fields]) OR "cardio pulmonary resuscitation"[All Fields])) OR "cardiopulmonary resuscitation"[MeSH Terms]) OR ("cardiopulmonary resuscitation"[MeSH Terms] OR ("cardiopulmonary"[All Fields] AND "resuscitation"[All Fields]) OR "cardiopulmonary resuscitation"[All Fields])) OR "resuscitation"[MeSH Terms]) OR ("resuscitation"[MeSH Terms] OR "resuscitation"[All Fields])) OR (("thorax"[MeSH Terms] OR "thorax"[All Fields] OR "chest"[All Fields]) AND compression[All Fields]))) AND ((((((("space flight"[MeSH Terms] OR ("space flight"[MeSH Terms] OR ("space"[All Fields] AND "flight"[All Fields]) OR "space flight"[All Fields])) OR "weightlessness"[MeSH Terms]) OR ("weightlessness"[MeSH Terms] OR "weightlessness"[All Fields] OR "microgravity"[All Fields])) OR "spacecraft"[MeSH Terms]) OR ("spacecraft"[MeSH Terms] OR "spacecraft"[All Fields])) OR "aerospace medicine"[MeSH Terms]) OR ("aerospace medicine"[MeSH Terms] OR ("aerospace"[All Fields] AND "medicine"[All Fields]) OR "aerospace medicine"[All Fields])) | 9 |
| 7 | What is the risk of musculoskeletal injury performing manual CPR after prolonged exposure to microgravity? (E.g. are current onboard exercise regimes sufficient to maintain required muscle strength and stability for safe and effective CPR without causing injury) | (("wounds and injuries"[MeSH Terms] OR ("wounds and injuries"[MeSH Terms] OR ("wounds"[All Fields] AND "injuries"[All Fields]) OR "wounds and injuries"[All Fields] OR "injury"[All Fields])) AND (((((((("cardiopulmonary resuscitation"[MeSH Terms] OR ("cardiopulmonary resuscitation"[MeSH Terms] OR ("cardiopulmonary"[All Fields] AND "resuscitation"[All Fields]) OR "cardiopulmonary resuscitation"[All Fields] OR "cpr"[All Fields])) OR "cardiopulmonary resuscitation"[MeSH Terms]) OR ("cardiopulmonary resuscitation"[MeSH Terms] OR ("cardiopulmonary"[All Fields] AND "resuscitation"[All Fields]) OR "cardiopulmonary resuscitation"[All Fields] OR ("cardio"[All Fields] AND "pulmonary"[All Fields] AND "resuscitation"[All Fields]) OR "cardio pulmonary resuscitation"[All Fields])) OR "cardiopulmonary resuscitation"[MeSH Terms]) OR ("cardiopulmonary resuscitation"[MeSH Terms] OR ("cardiopulmonary"[All Fields] AND "resuscitation"[All Fields]) OR "cardiopulmonary resuscitation"[All Fields])) OR "resuscitation"[MeSH Terms]) OR ("resuscitation"[MeSH Terms] OR "resuscitation"[All Fields])) OR (("thorax"[MeSH Terms] OR "thorax"[All Fields] OR "chest"[All Fields]) AND compression[All Fields]))) AND ((((((("space flight"[MeSH Terms] OR ("space flight"[MeSH Terms] OR ("space"[All Fields] AND "flight"[All Fields]) OR "space flight"[All Fields])) OR "weightlessness"[MeSH Terms]) OR ("weightlessness"[MeSH Terms] OR "weightlessness"[All Fields] OR "microgravity"[All Fields])) OR "spacecraft"[MeSH Terms]) OR ("spacecraft"[MeSH Terms] OR "spacecraft"[All Fields])) OR "aerospace medicine"[MeSH Terms]) OR ("aerospace medicine"[MeSH Terms] OR ("aerospace"[All Fields] AND "medicine"[All Fields]) OR "aerospace medicine"[All Fields])) | 33 |
| 8 | Does the chest compression technique (HS, ER, RBH) influence the feasibility of gaining iv-access? | ((((("infusions, intravenous"[MeSH Terms] OR ("infusions, intravenous"[MeSH Terms] OR ("infusions"[All Fields] AND "intravenous"[All Fields]) OR "intravenous infusions"[All Fields] OR ("intravenous"[All Fields] AND "infusion"[All Fields]) OR "intravenous infusion"[All Fields])) OR (intravenous[All Fields] AND access[All Fields])) OR "injections, intravenous"[MeSH Terms]) OR ("injections, intravenous"[MeSH Terms] OR ("injections"[All Fields] AND "intravenous"[All Fields]) OR "intravenous injections"[All Fields] OR ("intravenous"[All Fields] AND "injection"[All Fields]) OR "intravenous injection"[All Fields])) AND (((((((("cardiopulmonary resuscitation"[MeSH Terms] OR ("cardiopulmonary resuscitation"[MeSH Terms] OR ("cardiopulmonary"[All Fields] AND "resuscitation"[All Fields]) OR "cardiopulmonary resuscitation"[All Fields] OR "cpr"[All Fields])) OR "cardiopulmonary resuscitation"[MeSH Terms]) OR ("cardiopulmonary resuscitation"[MeSH Terms] OR ("cardiopulmonary"[All Fields] AND "resuscitation"[All Fields]) OR "cardiopulmonary resuscitation"[All Fields] OR ("cardio"[All Fields] AND "pulmonary"[All Fields] AND "resuscitation"[All Fields]) OR "cardio pulmonary resuscitation"[All Fields])) OR "cardiopulmonary resuscitation"[MeSH Terms]) OR ("cardiopulmonary resuscitation"[MeSH Terms] OR ("cardiopulmonary"[All Fields] AND "resuscitation"[All Fields]) OR "cardiopulmonary resuscitation"[All Fields])) OR "resuscitation"[MeSH Terms]) OR ("resuscitation"[MeSH Terms] OR "resuscitation"[All Fields])) OR (("thorax"[MeSH Terms] OR "thorax"[All Fields] OR "chest"[All Fields]) AND compression[All Fields]))) AND ((((((("space flight"[MeSH Terms] OR ("space flight"[MeSH Terms] OR ("space"[All Fields] AND "flight"[All Fields]) OR "space flight"[All Fields])) OR "weightlessness"[MeSH Terms]) OR ("weightlessness"[MeSH Terms] OR "weightlessness"[All Fields] OR "microgravity"[All Fields])) OR "spacecraft"[MeSH Terms]) OR ("spacecraft"[MeSH Terms] OR "spacecraft"[All Fields])) OR "aerospace medicine"[MeSH Terms]) OR ("aerospace medicine"[MeSH Terms] OR ("aerospace"[All Fields] AND "medicine"[All Fields]) OR "aerospace medicine"[All Fields])) | 2 |
| 9 | Does the chest compression technique (HS, ER, RBH) influence the feasibility of securing the airway? | ((((("intubation, intratracheal"[MeSH Terms] OR ("intubation"[MeSH Terms] OR "intubation"[All Fields])) OR "intubation, intratracheal"[MeSH Terms]) OR "airway management"[MeSH Terms]) OR airway[All Fields]) AND (((((((("cardiopulmonary resuscitation"[MeSH Terms] OR ("cardiopulmonary resuscitation"[MeSH Terms] OR ("cardiopulmonary"[All Fields] AND "resuscitation"[All Fields]) OR "cardiopulmonary resuscitation"[All Fields] OR "cpr"[All Fields])) OR "cardiopulmonary resuscitation"[MeSH Terms]) OR ("cardiopulmonary resuscitation"[MeSH Terms] OR ("cardiopulmonary"[All Fields] AND "resuscitation"[All Fields]) OR "cardiopulmonary resuscitation"[All Fields] OR ("cardio"[All Fields] AND "pulmonary"[All Fields] AND "resuscitation"[All Fields]) OR "cardio pulmonary resuscitation"[All Fields])) OR "cardiopulmonary resuscitation"[MeSH Terms]) OR ("cardiopulmonary resuscitation"[MeSH Terms] OR ("cardiopulmonary"[All Fields] AND "resuscitation"[All Fields]) OR "cardiopulmonary resuscitation"[All Fields])) OR "resuscitation"[MeSH Terms]) OR ("resuscitation"[MeSH Terms] OR "resuscitation"[All Fields])) OR (("thorax"[MeSH Terms] OR "thorax"[All Fields] OR "chest"[All Fields]) AND compression[All Fields]))) AND ((((((("space flight"[MeSH Terms] OR ("space flight"[MeSH Terms] OR ("space"[All Fields] AND "flight"[All Fields]) OR "space flight"[All Fields])) OR "weightlessness"[MeSH Terms]) OR ("weightlessness"[MeSH Terms] OR "weightlessness"[All Fields] OR "microgravity"[All Fields])) OR "spacecraft"[MeSH Terms]) OR ("spacecraft"[MeSH Terms] OR "spacecraft"[All Fields])) OR "aerospace medicine"[MeSH Terms]) OR ("aerospace medicine"[MeSH Terms] OR ("aerospace"[All Fields] AND "medicine"[All Fields]) OR "aerospace medicine"[All Fields])) | 45 |
| 10 | Does the HS-technique compared to the ER-technique accomplish better compressions depths in patients in cardiac arrest in microgravity? | ((((("weightlessness"[MeSH Terms] OR "weightlessness"[All Fields] OR "microgravity"[All Fields]) OR "weightlessness"[MeSH Terms]) OR ("weightlessness"[MeSH Terms] OR "weightlessness"[All Fields])) OR "weightlessness"[MeSH Terms]) OR "space flight"[MeSH Terms]) AND (((((("cardiopulmonary resuscitation"[MeSH Terms] OR ("cardiopulmonary"[All Fields] AND "resuscitation"[All Fields]) OR "cardiopulmonary resuscitation"[All Fields] OR "cpr"[All Fields]) OR "cardiopulmonary resuscitation"[MeSH Terms]) OR ("cardiopulmonary resuscitation"[MeSH Terms] OR ("cardiopulmonary"[All Fields] AND "resuscitation"[All Fields]) OR "cardiopulmonary resuscitation"[All Fields])) OR "cardiopulmonary resuscitation"[MeSH Terms]) OR ("resuscitation"[MeSH Terms] OR "resuscitation"[All Fields])) OR "resuscitation"[MeSH Terms]) | 34 |
| 11 | Does the HS-technique compared to the ER-technique accomplish a better cardiac output during CPR in patients in cardiac arrest in microgravity? | ((((("weightlessness"[MeSH Terms] OR "weightlessness"[All Fields] OR "microgravity"[All Fields]) OR "weightlessness"[MeSH Terms]) OR ("weightlessness"[MeSH Terms] OR "weightlessness"[All Fields])) OR "weightlessness"[MeSH Terms]) OR "space flight"[MeSH Terms]) AND (((((("cardiopulmonary resuscitation"[MeSH Terms] OR ("cardiopulmonary"[All Fields] AND "resuscitation"[All Fields]) OR "cardiopulmonary resuscitation"[All Fields] OR "cpr"[All Fields]) OR "cardiopulmonary resuscitation"[MeSH Terms]) OR ("cardiopulmonary resuscitation"[MeSH Terms] OR ("cardiopulmonary"[All Fields] AND "resuscitation"[All Fields]) OR "cardiopulmonary resuscitation"[All Fields])) OR "cardiopulmonary resuscitation"[MeSH Terms]) OR ("resuscitation"[MeSH Terms] OR "resuscitation"[All Fields])) OR "resuscitation"[MeSH Terms]) | 34 |
| 12 | Can the HS-technique compared to the ER-technique be performed more consistent regarding compression frequency during CPR in patients in cardiac arrest in microgravity? | ((((("weightlessness"[MeSH Terms] OR "weightlessness"[All Fields] OR "microgravity"[All Fields]) OR "weightlessness"[MeSH Terms]) OR ("weightlessness"[MeSH Terms] OR "weightlessness"[All Fields])) OR "weightlessness"[MeSH Terms]) OR "space flight"[MeSH Terms]) AND (((((("cardiopulmonary resuscitation"[MeSH Terms] OR ("cardiopulmonary"[All Fields] AND "resuscitation"[All Fields]) OR "cardiopulmonary resuscitation"[All Fields] OR "cpr"[All Fields]) OR "cardiopulmonary resuscitation"[MeSH Terms]) OR ("cardiopulmonary resuscitation"[MeSH Terms] OR ("cardiopulmonary"[All Fields] AND "resuscitation"[All Fields]) OR "cardiopulmonary resuscitation"[All Fields])) OR "cardiopulmonary resuscitation"[MeSH Terms]) OR ("resuscitation"[MeSH Terms] OR "resuscitation"[All Fields])) OR "resuscitation"[MeSH Terms]) | 34 |
| 13 | Should a patient in cardiac arrest in microgravity be fastened on the crew medical restraint system for CPR or should he be free floating regarding feasibility of CPR? | ((("restraint, physical"[MeSH Terms] OR restraint[All Fields]) OR "restraint, physical"[MeSH Terms]) OR fasten[All Fields]) AND (((((("weightlessness"[MeSH Terms] OR "weightlessness"[All Fields] OR "microgravity"[All Fields]) OR "weightlessness"[MeSH Terms]) OR ("weightlessness"[MeSH Terms] OR "weightlessness"[All Fields])) OR "weightlessness"[MeSH Terms]) OR "space flight"[MeSH Terms]) AND (((((("cardiopulmonary resuscitation"[MeSH Terms] OR ("cardiopulmonary"[All Fields] AND "resuscitation"[All Fields]) OR "cardiopulmonary resuscitation"[All Fields] OR "cpr"[All Fields]) OR "cardiopulmonary resuscitation"[MeSH Terms]) OR cardiopulmonary[All Fields]) OR "cardiopulmonary resuscitation"[MeSH Terms]) OR ("resuscitation"[MeSH Terms] OR "resuscitation"[All Fields])) OR "cardiopulmonary resuscitation"[MeSH Terms])) | 30 |
| 14 | Should a patient in cardiac arrest in microgravity be fastened on the crew medical restraint system for CPR or should he be free floating regarding beginning of effective chest compressions? | ((("restraint, physical"[MeSH Terms] OR restraint[All Fields]) OR "restraint, physical"[MeSH Terms]) OR fasten[All Fields]) AND (((((("weightlessness"[MeSH Terms] OR "weightlessness"[All Fields] OR "microgravity"[All Fields]) OR "weightlessness"[MeSH Terms]) OR ("weightlessness"[MeSH Terms] OR "weightlessness"[All Fields])) OR "weightlessness"[MeSH Terms]) OR "space flight"[MeSH Terms]) AND (((((("cardiopulmonary resuscitation"[MeSH Terms] OR ("cardiopulmonary"[All Fields] AND "resuscitation"[All Fields]) OR "cardiopulmonary resuscitation"[All Fields] OR "cpr"[All Fields]) OR "cardiopulmonary resuscitation"[MeSH Terms]) OR cardiopulmonary[All Fields]) OR "cardiopulmonary resuscitation"[MeSH Terms]) OR ("resuscitation"[MeSH Terms] OR "resuscitation"[All Fields])) OR "cardiopulmonary resuscitation"[MeSH Terms])) | 30 |
| 15 | Does an automated chest compression device have an advantage over manual chest compressions (HS-technique) regarding compression depth during CPR in patients in cardiac arrest in microgravity? | ((((("weightlessness"[MeSH Terms] OR "weightlessness"[All Fields] OR "microgravity"[All Fields]) OR "weightlessness"[MeSH Terms]) OR ("weightlessness"[MeSH Terms] OR "weightlessness"[All Fields])) OR "weightlessness"[MeSH Terms]) OR "space flight"[MeSH Terms]) AND (((((("cardiopulmonary resuscitation"[MeSH Terms] OR ("cardiopulmonary"[All Fields] AND "resuscitation"[All Fields]) OR "cardiopulmonary resuscitation"[All Fields] OR "cpr"[All Fields]) OR "cardiopulmonary resuscitation"[MeSH Terms]) OR ("cardiopulmonary resuscitation"[MeSH Terms] OR ("cardiopulmonary"[All Fields] AND "resuscitation"[All Fields]) OR "cardiopulmonary resuscitation"[All Fields])) OR "cardiopulmonary resuscitation"[MeSH Terms]) OR ("resuscitation"[MeSH Terms] OR "resuscitation"[All Fields])) OR "resuscitation"[MeSH Terms])resuscitation"[MeSH Terms]) OR ("resuscitation"[MeSH Terms] OR "resuscitation"[All Fields])) OR "cardiopulmonary resuscitation"[MeSH Terms]) | 34 |
| 16 | Does an automated chest compression device have an advantage over manual chest compressions (HS-technique) regarding cardiac output during CPR in patients with cardiac arrest in microgravity? | ((((("weightlessness"[MeSH Terms] OR "weightlessness"[All Fields] OR "microgravity"[All Fields]) OR "weightlessness"[MeSH Terms]) OR ("weightlessness"[MeSH Terms] OR "weightlessness"[All Fields])) OR "weightlessness"[MeSH Terms]) OR "space flight"[MeSH Terms]) AND (((((("cardiopulmonary resuscitation"[MeSH Terms] OR ("cardiopulmonary"[All Fields] AND "resuscitation"[All Fields]) OR "cardiopulmonary resuscitation"[All Fields] OR "cpr"[All Fields]) OR "cardiopulmonary resuscitation"[MeSH Terms]) OR ("cardiopulmonary resuscitation"[MeSH Terms] OR ("cardiopulmonary"[All Fields] AND "resuscitation"[All Fields]) OR "cardiopulmonary resuscitation"[All Fields])) OR "cardiopulmonary resuscitation"[MeSH Terms]) OR ("resuscitation"[MeSH Terms] OR "resuscitation"[All Fields])) OR "resuscitation"[MeSH Terms]) | 34 |
| 17 | Does an automated chest compression device have an advantage over manual chest compressions (HS-technique) regarding consistency of compression frequency during CPR in patients with cardiac arrest in microgravity? | ((((("weightlessness"[MeSH Terms] OR "weightlessness"[All Fields] OR "microgravity"[All Fields]) OR "weightlessness"[MeSH Terms]) OR ("weightlessness"[MeSH Terms] OR "weightlessness"[All Fields])) OR "weightlessness"[MeSH Terms]) OR "space flight"[MeSH Terms]) AND (((((("cardiopulmonary resuscitation"[MeSH Terms] OR ("cardiopulmonary"[All Fields] AND "resuscitation"[All Fields]) OR "cardiopulmonary resuscitation"[All Fields] OR "cpr"[All Fields]) OR "cardiopulmonary resuscitation"[MeSH Terms]) OR ("cardiopulmonary resuscitation"[MeSH Terms] OR ("cardiopulmonary"[All Fields] AND "resuscitation"[All Fields]) OR "cardiopulmonary resuscitation"[All Fields])) OR "cardiopulmonary resuscitation"[MeSH Terms]) OR ("resuscitation"[MeSH Terms] OR "resuscitation"[All Fields])) OR "resuscitation"[MeSH Terms]) | 34 |
| 18 | Can an automated chest compression device be applied to a patient in cardiac arrest in microgravity in an adequate amount of time compared to CPR in the HS-technique? | ((((("weightlessness"[MeSH Terms] OR "weightlessness"[All Fields] OR "microgravity"[All Fields]) OR "weightlessness"[MeSH Terms]) OR ("weightlessness"[MeSH Terms] OR "weightlessness"[All Fields])) OR "weightlessness"[MeSH Terms]) OR "space flight"[MeSH Terms]) AND (((((("cardiopulmonary resuscitation"[MeSH Terms] OR ("cardiopulmonary"[All Fields] AND "resuscitation"[All Fields]) OR "cardiopulmonary resuscitation"[All Fields] OR "cpr"[All Fields]) OR "cardiopulmonary resuscitation"[MeSH Terms]) OR ("cardiopulmonary resuscitation"[MeSH Terms] OR ("cardiopulmonary"[All Fields] AND "resuscitation"[All Fields]) OR "cardiopulmonary resuscitation"[All Fields])) OR "cardiopulmonary resuscitation"[MeSH Terms]) OR ("resuscitation"[MeSH Terms] OR "resuscitation"[All Fields])) OR "resuscitation"[MeSH Terms]) | 34 |
| 19 | Can an automated chest compression device be operated effectively while being used on a patient in cardiac arrest free floating in microgravity? | ((((("weightlessness"[MeSH Terms] OR "weightlessness"[All Fields] OR "microgravity"[All Fields]) OR "weightlessness"[MeSH Terms]) OR ("weightlessness"[MeSH Terms] OR "weightlessness"[All Fields])) OR "weightlessness"[MeSH Terms]) OR "space flight"[MeSH Terms]) AND (((((("cardiopulmonary resuscitation"[MeSH Terms] OR ("cardiopulmonary"[All Fields] AND "resuscitation"[All Fields]) OR "cardiopulmonary resuscitation"[All Fields] OR "cpr"[All Fields]) OR "cardiopulmonary resuscitation"[MeSH Terms]) OR ("cardiopulmonary resuscitation"[MeSH Terms] OR ("cardiopulmonary"[All Fields] AND "resuscitation"[All Fields]) OR "cardiopulmonary resuscitation"[All Fields])) OR "cardiopulmonary resuscitation"[MeSH Terms]) OR ("resuscitation"[MeSH Terms] OR "resuscitation"[All Fields])) OR "resuscitation"[MeSH Terms]) | 34 |
| 20 | Can an automated chest compression device be operated safely while being used on a patient in cardiac arrest free floating in microgravity? | ((((("weightlessness"[MeSH Terms] OR "weightlessness"[All Fields] OR "microgravity"[All Fields]) OR "weightlessness"[MeSH Terms]) OR ("weightlessness"[MeSH Terms] OR "weightlessness"[All Fields])) OR "weightlessness"[MeSH Terms]) OR "space flight"[MeSH Terms]) AND (((((("cardiopulmonary resuscitation"[MeSH Terms] OR ("cardiopulmonary"[All Fields] AND "resuscitation"[All Fields]) OR "cardiopulmonary resuscitation"[All Fields] OR "cpr"[All Fields]) OR "cardiopulmonary resuscitation"[MeSH Terms]) OR ("cardiopulmonary resuscitation"[MeSH Terms] OR ("cardiopulmonary"[All Fields] AND "resuscitation"[All Fields]) OR "cardiopulmonary resuscitation"[All Fields])) OR "cardiopulmonary resuscitation"[MeSH Terms]) OR ("resuscitation"[MeSH Terms] OR "resuscitation"[All Fields])) OR "resuscitation"[MeSH Terms]) | 34 |
| 21 | Can an automated chest compression device be operated effectively while being used on a patient in cardiac arrest strapped to the restraint system in microgravity? | ((((("weightlessness"[MeSH Terms] OR "weightlessness"[All Fields] OR "microgravity"[All Fields]) OR "weightlessness"[MeSH Terms]) OR ("weightlessness"[MeSH Terms] OR "weightlessness"[All Fields])) OR "weightlessness"[MeSH Terms]) OR "space flight"[MeSH Terms]) AND (((((("cardiopulmonary resuscitation"[MeSH Terms] OR ("cardiopulmonary"[All Fields] AND "resuscitation"[All Fields]) OR "cardiopulmonary resuscitation"[All Fields] OR "cpr"[All Fields]) OR "cardiopulmonary resuscitation"[MeSH Terms]) OR ("cardiopulmonary resuscitation"[MeSH Terms] OR ("cardiopulmonary"[All Fields] AND "resuscitation"[All Fields]) OR "cardiopulmonary resuscitation"[All Fields])) OR "cardiopulmonary resuscitation"[MeSH Terms]) OR ("resuscitation"[MeSH Terms] OR "resuscitation"[All Fields])) OR "resuscitation"[MeSH Terms]) | 34 |
| 22 | Can an automated chest compression device be operated safely while being used on a patient in cardiac arrest strapped to the restraint system in microgravity? | ((((("weightlessness"[MeSH Terms] OR "weightlessness"[All Fields] OR "microgravity"[All Fields]) OR "weightlessness"[MeSH Terms]) OR ("weightlessness"[MeSH Terms] OR "weightlessness"[All Fields])) OR "weightlessness"[MeSH Terms]) OR "space flight"[MeSH Terms]) AND (((((("cardiopulmonary resuscitation"[MeSH Terms] OR ("cardiopulmonary"[All Fields] AND "resuscitation"[All Fields]) OR "cardiopulmonary resuscitation"[All Fields] OR "cpr"[All Fields]) OR "cardiopulmonary resuscitation"[MeSH Terms]) OR ("cardiopulmonary resuscitation"[MeSH Terms] OR ("cardiopulmonary"[All Fields] AND "resuscitation"[All Fields]) OR "cardiopulmonary resuscitation"[All Fields])) OR "cardiopulmonary resuscitation"[MeSH Terms]) OR ("resuscitation"[MeSH Terms] OR "resuscitation"[All Fields])) OR "resuscitation"[MeSH Terms]) | 34 |
| 23 | Is it reasonable to transport an 8,0 kg heavy automated chest compression device on a space mission compared to the lack of this device for use in CPR of a patient in cardiac arrest in microgravity? | ((((("weightlessness"[MeSH Terms] OR "weightlessness"[All Fields] OR "microgravity"[All Fields]) OR "weightlessness"[MeSH Terms]) OR ("weightlessness"[MeSH Terms] OR "weightlessness"[All Fields])) OR "weightlessness"[MeSH Terms]) OR "space flight"[MeSH Terms]) AND (((((("cardiopulmonary resuscitation"[MeSH Terms] OR ("cardiopulmonary"[All Fields] AND "resuscitation"[All Fields]) OR "cardiopulmonary resuscitation"[All Fields] OR "cpr"[All Fields]) OR "cardiopulmonary resuscitation"[MeSH Terms]) OR ("cardiopulmonary resuscitation"[MeSH Terms] OR ("cardiopulmonary"[All Fields] AND "resuscitation"[All Fields]) OR "cardiopulmonary resuscitation"[All Fields])) OR "cardiopulmonary resuscitation"[MeSH Terms]) OR ("resuscitation"[MeSH Terms] OR "resuscitation"[All Fields])) OR "resuscitation"[MeSH Terms]) | 34 |
| 24 | Is the endotracheal laryngoscopy-guided intubation superior compared to the use of a supraglottic airway device (SGA) regarding the time to establish the airway during CPR in a patient with cardiac arrest in microgravity? | ((((("weightlessness"[MeSH Terms] OR "weightlessness"[All Fields] OR "microgravity"[All Fields]) OR "weightlessness"[MeSH Terms]) OR ("weightlessness"[MeSH Terms] OR "weightlessness"[All Fields])) OR "weightlessness"[MeSH Terms]) OR "space flight"[MeSH Terms]) AND ((((((((((("intubation"[MeSH Terms] OR "intubation"[All Fields]) OR "intubation"[MeSH Terms]) OR endotracheal[All Fields]) OR "intubation, intratracheal"[MeSH Terms]) OR "airway management"[MeSH Terms]) OR airway[All Fields]) OR "laryngeal masks"[MeSH Terms]) OR ("laryngeal masks"[MeSH Terms] OR ("laryngeal"[All Fields] AND "masks"[All Fields]) OR "laryngeal masks"[All Fields] OR ("laryngeal"[All Fields] AND "mask"[All Fields]) OR "laryngeal mask"[All Fields])) OR combitube[All Fields]) OR (("larynx"[MeSH Terms] OR "larynx"[All Fields] OR "laryngeal"[All Fields]) AND tube[All Fields])) OR i-gel[All Fields]) | 55 |
| 25 | Is the endotracheal laryngoscopy-guided intubation superior compared to the use of a SGA regarding the training time of the care provider during CPR in a patient with cardiac arrest in microgravity? | ((((("weightlessness"[MeSH Terms] OR "weightlessness"[All Fields] OR "microgravity"[All Fields]) OR "weightlessness"[MeSH Terms]) OR ("weightlessness"[MeSH Terms] OR "weightlessness"[All Fields])) OR "weightlessness"[MeSH Terms]) OR "space flight"[MeSH Terms]) AND ((((((((((("intubation"[MeSH Terms] OR "intubation"[All Fields]) OR "intubation"[MeSH Terms]) OR endotracheal[All Fields]) OR "intubation, intratracheal"[MeSH Terms]) OR "airway management"[MeSH Terms]) OR airway[All Fields]) OR "laryngeal masks"[MeSH Terms]) OR ("laryngeal masks"[MeSH Terms] OR ("laryngeal"[All Fields] AND "masks"[All Fields]) OR "laryngeal masks"[All Fields] OR ("laryngeal"[All Fields] AND "mask"[All Fields]) OR "laryngeal mask"[All Fields])) OR combitube[All Fields]) OR (("larynx"[MeSH Terms] OR "larynx"[All Fields] OR "laryngeal"[All Fields]) AND tube[All Fields])) OR i-gel[All Fields]) | 55 |
| 26 | Is the endotracheal laryngoscopy-guided intubation superior compared to the use of a SGA regarding the success rate of correct insertion during CPR in a patient with cardiac arrest in microgravity? | ((((("weightlessness"[MeSH Terms] OR "weightlessness"[All Fields] OR "microgravity"[All Fields]) OR "weightlessness"[MeSH Terms]) OR ("weightlessness"[MeSH Terms] OR "weightlessness"[All Fields])) OR "weightlessness"[MeSH Terms]) OR "space flight"[MeSH Terms]) AND ((((((((((("intubation"[MeSH Terms] OR "intubation"[All Fields]) OR "intubation"[MeSH Terms]) OR endotracheal[All Fields]) OR "intubation, intratracheal"[MeSH Terms]) OR "airway management"[MeSH Terms]) OR airway[All Fields]) OR "laryngeal masks"[MeSH Terms]) OR ("laryngeal masks"[MeSH Terms] OR ("laryngeal"[All Fields] AND "masks"[All Fields]) OR "laryngeal masks"[All Fields] OR ("laryngeal"[All Fields] AND "mask"[All Fields]) OR "laryngeal mask"[All Fields])) OR combitube[All Fields]) OR (("larynx"[MeSH Terms] OR "larynx"[All Fields] OR "laryngeal"[All Fields]) AND tube[All Fields])) OR i-gel[All Fields]) | 55 |
| 27 | Is the use of a SGA superior to the use of bag-mask-ventilation regarding time to establish the airway during CPR in a patient with cardiac arrest in microgravity? | ((((("weightlessness"[MeSH Terms] OR "weightlessness"[All Fields] OR "microgravity"[All Fields]) OR "weightlessness"[MeSH Terms]) OR ("weightlessness"[MeSH Terms] OR "weightlessness"[All Fields])) OR "weightlessness"[MeSH Terms]) OR "space flight"[MeSH Terms]) AND ((((((((("airway management"[MeSH Terms] OR airway[All Fields]) OR "laryngeal masks"[MeSH Terms]) OR ("laryngeal masks"[MeSH Terms] OR ("laryngeal"[All Fields] AND "masks"[All Fields]) OR "laryngeal masks"[All Fields] OR ("laryngeal"[All Fields] AND "mask"[All Fields]) OR "laryngeal mask"[All Fields])) OR combitube[All Fields]) OR (("larynx"[MeSH Terms] OR "larynx"[All Fields] OR "laryngeal"[All Fields]) AND tube[All Fields])) OR i-gel[All Fields]) OR (bag[All Fields] AND ("masks"[MeSH Terms] OR "masks"[All Fields] OR "mask"[All Fields]) AND ("ventilation"[MeSH Terms] OR "ventilation"[All Fields] OR "respiration"[MeSH Terms] OR "respiration"[All Fields]))) OR bag-mask-ventilation[All Fields]) OR bvm[All Fields]) | 54 |
| 28 | Is the use of a SGA superior to the use of bag-mask-ventilation regarding the training time of the care provider during CPR in a patient with cardiac arrest in microgravity? | ((((("weightlessness"[MeSH Terms] OR "weightlessness"[All Fields] OR "microgravity"[All Fields]) OR "weightlessness"[MeSH Terms]) OR ("weightlessness"[MeSH Terms] OR "weightlessness"[All Fields])) OR "weightlessness"[MeSH Terms]) OR "space flight"[MeSH Terms]) AND ((((((((("airway management"[MeSH Terms] OR airway[All Fields]) OR "laryngeal masks"[MeSH Terms]) OR ("laryngeal masks"[MeSH Terms] OR ("laryngeal"[All Fields] AND "masks"[All Fields]) OR "laryngeal masks"[All Fields] OR ("laryngeal"[All Fields] AND "mask"[All Fields]) OR "laryngeal mask"[All Fields])) OR combitube[All Fields]) OR (("larynx"[MeSH Terms] OR "larynx"[All Fields] OR "laryngeal"[All Fields]) AND tube[All Fields])) OR i-gel[All Fields]) OR (bag[All Fields] AND ("masks"[MeSH Terms] OR "masks"[All Fields] OR "mask"[All Fields]) AND ("ventilation"[MeSH Terms] OR "ventilation"[All Fields] OR "respiration"[MeSH Terms] OR "respiration"[All Fields]))) OR bag-mask-ventilation[All Fields]) OR bvm[All Fields]) | 54 |
| 29 | Is the use of a SGA superior to the use of bag-mask-ventilation regarding the success rate of adequate ventilation during CPR in a patient with cardiac arrest in microgravity? | ((((("weightlessness"[MeSH Terms] OR "weightlessness"[All Fields] OR "microgravity"[All Fields]) OR "weightlessness"[MeSH Terms]) OR ("weightlessness"[MeSH Terms] OR "weightlessness"[All Fields])) OR "weightlessness"[MeSH Terms]) OR "space flight"[MeSH Terms]) AND ((((((((("airway management"[MeSH Terms] OR airway[All Fields]) OR "laryngeal masks"[MeSH Terms]) OR ("laryngeal masks"[MeSH Terms] OR ("laryngeal"[All Fields] AND "masks"[All Fields]) OR "laryngeal masks"[All Fields] OR ("laryngeal"[All Fields] AND "mask"[All Fields]) OR "laryngeal mask"[All Fields])) OR combitube[All Fields]) OR (("larynx"[MeSH Terms] OR "larynx"[All Fields] OR "laryngeal"[All Fields]) AND tube[All Fields])) OR i-gel[All Fields]) OR (bag[All Fields] AND ("masks"[MeSH Terms] OR "masks"[All Fields] OR "mask"[All Fields]) AND ("ventilation"[MeSH Terms] OR "ventilation"[All Fields] OR "respiration"[MeSH Terms] OR "respiration"[All Fields]))) OR bag-mask-ventilation[All Fields]) OR bvm[All Fields]) | 54 |
| 30 | Is the use of a SGA superior to the use of bag-mask-ventilation regarding leakage during CPR in a patient with cardiac arrest in microgravity? | ((((("weightlessness"[MeSH Terms] OR "weightlessness"[All Fields] OR "microgravity"[All Fields]) OR "weightlessness"[MeSH Terms]) OR ("weightlessness"[MeSH Terms] OR "weightlessness"[All Fields])) OR "weightlessness"[MeSH Terms]) OR "space flight"[MeSH Terms]) AND ((((((((("airway management"[MeSH Terms] OR airway[All Fields]) OR "laryngeal masks"[MeSH Terms]) OR ("laryngeal masks"[MeSH Terms] OR ("laryngeal"[All Fields] AND "masks"[All Fields]) OR "laryngeal masks"[All Fields] OR ("laryngeal"[All Fields] AND "mask"[All Fields]) OR "laryngeal mask"[All Fields])) OR combitube[All Fields]) OR (("larynx"[MeSH Terms] OR "larynx"[All Fields] OR "laryngeal"[All Fields]) AND tube[All Fields])) OR i-gel[All Fields]) OR (bag[All Fields] AND ("masks"[MeSH Terms] OR "masks"[All Fields] OR "mask"[All Fields]) AND ("ventilation"[MeSH Terms] OR "ventilation"[All Fields] OR "respiration"[MeSH Terms] OR "respiration"[All Fields]))) OR bag-mask-ventilation[All Fields]) OR bvm[All Fields]) | 54 |
| 31 | Is the free floating position superior to the restrained position regarding laryngoscopy-guided endotracheal intubation in a patient in cardiac arrest in microgravity? | ((((("weightlessness"[MeSH Terms] OR "weightlessness"[All Fields] OR "microgravity"[All Fields]) OR "weightlessness"[MeSH Terms]) OR ("weightlessness"[MeSH Terms] OR "weightlessness"[All Fields])) OR "weightlessness"[MeSH Terms]) OR "space flight"[MeSH Terms]) AND (((((((("intubation"[MeSH Terms] OR "intubation"[All Fields]) OR "intubation"[MeSH Terms]) OR endotracheal[All Fields]) OR "intubation, intratracheal"[MeSH Terms]) OR "airway management"[MeSH Terms]) OR airway[All Fields]) OR "laryngoscopy"[MeSH Terms]) OR ("laryngoscopy"[MeSH Terms] OR "laryngoscopy"[All Fields])) | 56 |
| 32 | Is the free floating position superior to the restrained position while performing laryngoscopy-guided endotracheal intubation regarding the time to establish the airway in a patient in cardiac arrest in microgravity? | ((((("weightlessness"[MeSH Terms] OR "weightlessness"[All Fields] OR "microgravity"[All Fields]) OR "weightlessness"[MeSH Terms]) OR ("weightlessness"[MeSH Terms] OR "weightlessness"[All Fields])) OR "weightlessness"[MeSH Terms]) OR "space flight"[MeSH Terms]) AND (((((((("intubation"[MeSH Terms] OR "intubation"[All Fields]) OR "intubation"[MeSH Terms]) OR endotracheal[All Fields]) OR "intubation, intratracheal"[MeSH Terms]) OR "airway management"[MeSH Terms]) OR airway[All Fields]) OR "laryngoscopy"[MeSH Terms]) OR ("laryngoscopy"[MeSH Terms] OR "laryngoscopy"[All Fields])) | 56 |
| 33 | Is the free floating position superior to the restrained position while performing laryngoscopy-guided endotracheal intubation regarding the training time of the care provider in a patient in cardiac arrest in microgravity? | ((((("weightlessness"[MeSH Terms] OR "weightlessness"[All Fields] OR "microgravity"[All Fields]) OR "weightlessness"[MeSH Terms]) OR ("weightlessness"[MeSH Terms] OR "weightlessness"[All Fields])) OR "weightlessness"[MeSH Terms]) OR "space flight"[MeSH Terms]) AND (((((((("intubation"[MeSH Terms] OR "intubation"[All Fields]) OR "intubation"[MeSH Terms]) OR endotracheal[All Fields]) OR "intubation, intratracheal"[MeSH Terms]) OR "airway management"[MeSH Terms]) OR airway[All Fields]) OR "laryngoscopy"[MeSH Terms]) OR ("laryngoscopy"[MeSH Terms] OR "laryngoscopy"[All Fields])) | 56 |
| 34 | Is the free floating position superior to the restrained position while performing laryngoscopy-guided endotracheal intubation regarding the success rate of correct insertion in a patient in cardiac arrest in microgravity? | ((((("weightlessness"[MeSH Terms] OR "weightlessness"[All Fields] OR "microgravity"[All Fields]) OR "weightlessness"[MeSH Terms]) OR ("weightlessness"[MeSH Terms] OR "weightlessness"[All Fields])) OR "weightlessness"[MeSH Terms]) OR "space flight"[MeSH Terms]) AND (((((((("intubation"[MeSH Terms] OR "intubation"[All Fields]) OR "intubation"[MeSH Terms]) OR endotracheal[All Fields]) OR "intubation, intratracheal"[MeSH Terms]) OR "airway management"[MeSH Terms]) OR airway[All Fields]) OR "laryngoscopy"[MeSH Terms]) OR ("laryngoscopy"[MeSH Terms] OR "laryngoscopy"[All Fields])) | 56 |
| 35 | Is the endotracheal video-laryngoscopy-guided intubation superior to the laryngoscopy-guided intubation regarding the time to establish the airway during CPR in a patient in cardiac arrest in microgravity? | ((((("weightlessness"[MeSH Terms] OR "weightlessness"[All Fields] OR "microgravity"[All Fields]) OR "weightlessness"[MeSH Terms]) OR ("weightlessness"[MeSH Terms] OR "weightlessness"[All Fields])) OR "weightlessness"[MeSH Terms]) OR "space flight"[MeSH Terms]) AND (((((((((((("intubation"[MeSH Terms] OR "intubation"[All Fields]) OR "intubation"[MeSH Terms]) OR endotracheal[All Fields]) OR "intubation, intratracheal"[MeSH Terms]) OR "airway management"[MeSH Terms]) OR airway[All Fields]) OR "laryngoscopy"[MeSH Terms]) OR ("laryngoscopy"[MeSH Terms] OR "laryngoscopy"[All Fields])) OR videolaryngoscopy[All Fields]) OR (("videotape recording"[MeSH Terms] OR ("videotape"[All Fields] AND "recording"[All Fields]) OR "videotape recording"[All Fields] OR "video"[All Fields]) AND ("laryngoscopy"[MeSH Terms] OR "laryngoscopy"[All Fields]))) OR ("bronchoscopy"[MeSH Terms] OR "bronchoscopy"[All Fields])) OR "bronchoscopy"[MeSH Terms]) | 56 |
| 36 | Is the endotracheal video-laryngoscopy-guided intubation superior to the laryngoscopy-guided intubation regarding success rate of correct insertion during CPR in a patient in cardiac arrest in microgravity? | ((((("weightlessness"[MeSH Terms] OR "weightlessness"[All Fields] OR "microgravity"[All Fields]) OR "weightlessness"[MeSH Terms]) OR ("weightlessness"[MeSH Terms] OR "weightlessness"[All Fields])) OR "weightlessness"[MeSH Terms]) OR "space flight"[MeSH Terms]) AND (((((((((((("intubation"[MeSH Terms] OR "intubation"[All Fields]) OR "intubation"[MeSH Terms]) OR endotracheal[All Fields]) OR "intubation, intratracheal"[MeSH Terms]) OR "airway management"[MeSH Terms]) OR airway[All Fields]) OR "laryngoscopy"[MeSH Terms]) OR ("laryngoscopy"[MeSH Terms] OR "laryngoscopy"[All Fields])) OR videolaryngoscopy[All Fields]) OR (("videotape recording"[MeSH Terms] OR ("videotape"[All Fields] AND "recording"[All Fields]) OR "videotape recording"[All Fields] OR "video"[All Fields]) AND ("laryngoscopy"[MeSH Terms] OR "laryngoscopy"[All Fields]))) OR ("bronchoscopy"[MeSH Terms] OR "bronchoscopy"[All Fields])) OR "bronchoscopy"[MeSH Terms]) | 56 |
| 37 | Is the endotracheal video-laryngoscopy-guided intubation superior to the laryngoscopy-guided intubation regarding the training time of the care provider during CPR in a patient in cardiac arrest in microgravity? | ((((("weightlessness"[MeSH Terms] OR "weightlessness"[All Fields] OR "microgravity"[All Fields]) OR "weightlessness"[MeSH Terms]) OR ("weightlessness"[MeSH Terms] OR "weightlessness"[All Fields])) OR "weightlessness"[MeSH Terms]) OR "space flight"[MeSH Terms]) AND (((((((((((("intubation"[MeSH Terms] OR "intubation"[All Fields]) OR "intubation"[MeSH Terms]) OR endotracheal[All Fields]) OR "intubation, intratracheal"[MeSH Terms]) OR "airway management"[MeSH Terms]) OR airway[All Fields]) OR "laryngoscopy"[MeSH Terms]) OR ("laryngoscopy"[MeSH Terms] OR "laryngoscopy"[All Fields])) OR videolaryngoscopy[All Fields]) OR (("videotape recording"[MeSH Terms] OR ("videotape"[All Fields] AND "recording"[All Fields]) OR "videotape recording"[All Fields] OR "video"[All Fields]) AND ("laryngoscopy"[MeSH Terms] OR "laryngoscopy"[All Fields]))) OR ("bronchoscopy"[MeSH Terms] OR "bronchoscopy"[All Fields])) OR "bronchoscopy"[MeSH Terms]) | 56 |
| 38 | Could microgravity increase the difficulty of laryngeal intubation? | ((((("weightlessness"[MeSH Terms] OR "weightlessness"[All Fields] OR "microgravity"[All Fields]) OR "weightlessness"[MeSH Terms]) OR ("weightlessness"[MeSH Terms] OR "weightlessness"[All Fields])) OR "weightlessness"[MeSH Terms]) OR "space flight"[MeSH Terms]) AND (((((((("intubation"[MeSH Terms] OR "intubation"[All Fields]) OR "intubation"[MeSH Terms]) OR endotracheal[All Fields]) OR "intubation, intratracheal"[MeSH Terms]) OR "airway management"[MeSH Terms]) OR airway[All Fields]) OR "laryngoscopy"[MeSH Terms]) OR ("laryngoscopy"[MeSH Terms] OR "laryngoscopy"[All Fields])) | 56 |
| 39 | Are the skills with laryngeal intubation at usual gravity sufficient for laryngeal intubation in microgravity? | ((((("weightlessness"[MeSH Terms] OR "weightlessness"[All Fields] OR "microgravity"[All Fields]) OR "weightlessness"[MeSH Terms]) OR ("weightlessness"[MeSH Terms] OR "weightlessness"[All Fields])) OR "weightlessness"[MeSH Terms]) OR "space flight"[MeSH Terms]) AND (((((((("intubation"[MeSH Terms] OR "intubation"[All Fields]) OR "intubation"[MeSH Terms]) OR endotracheal[All Fields]) OR "intubation, intratracheal"[MeSH Terms]) OR "airway management"[MeSH Terms]) OR airway[All Fields]) OR "laryngoscopy"[MeSH Terms]) OR ("laryngoscopy"[MeSH Terms] OR "laryngoscopy"[All Fields])) | 56 |
| 40 | Is an electrical suction unit more efficient regarding suction volume per minute compared to a manual suction unit during CPR in a patient with cardiac arrest in microgravity? | (("suction"[MeSH Terms] OR ("suction"[MeSH Terms] OR "suction"[All Fields])) OR aspiration[All Fields]) AND ((((("weightlessness"[MeSH Terms] OR "weightlessness"[All Fields] OR "microgravity"[All Fields]) OR "weightlessness"[MeSH Terms]) OR ("weightlessness"[MeSH Terms] OR "weightlessness"[All Fields])) OR "weightlessness"[MeSH Terms]) OR "space flight"[MeSH Terms]) | 38 |
| 41 | Is an electrical suction unit more efficient regarding simplicity of use compared to a manual suction unit during CPR in a patient with cardiac arrest in microgravity? | (("suction"[MeSH Terms] OR ("suction"[MeSH Terms] OR "suction"[All Fields])) OR aspiration[All Fields]) AND ((((("weightlessness"[MeSH Terms] OR "weightlessness"[All Fields] OR "microgravity"[All Fields]) OR "weightlessness"[MeSH Terms]) OR ("weightlessness"[MeSH Terms] OR "weightlessness"[All Fields])) OR "weightlessness"[MeSH Terms]) OR "space flight"[MeSH Terms]) | 38 |
| 42 | Is an electrical suction unit more efficient regarding contamination while suctioning compared to a manual suction unit during cpr in a patient with cardiac arrest in microgravity? | (("suction"[MeSH Terms] OR ("suction"[MeSH Terms] OR "suction"[All Fields])) OR aspiration[All Fields]) AND ((((("weightlessness"[MeSH Terms] OR "weightlessness"[All Fields] OR "microgravity"[All Fields]) OR "weightlessness"[MeSH Terms]) OR ("weightlessness"[MeSH Terms] OR "weightlessness"[All Fields])) OR "weightlessness"[MeSH Terms]) OR "space flight"[MeSH Terms]) | 38 |
| 43 | Is flammability in a space vehicle higher when ventilating a patient during CPR with 100% oxygen compared to ventilating the patient with room air with no additional gas extraction? | ((((((((((("cardiopulmonary resuscitation"[MeSH Terms] OR ("cardiopulmonary"[All Fields] AND "resuscitation"[All Fields]) OR "cardiopulmonary resuscitation"[All Fields] OR "cpr"[All Fields]) OR "cardiopulmonary resuscitation"[MeSH Terms]) OR cardiopulmonary[All Fields]) OR "cardiopulmonary resuscitation"[MeSH Terms]) OR ("resuscitation"[MeSH Terms] OR "resuscitation"[All Fields])) OR "cardiopulmonary resuscitation"[MeSH Terms]) OR "anesthesia"[MeSH Terms]) OR ("anaesthesia"[All Fields] OR "anesthesia"[MeSH Terms] OR "anesthesia"[All Fields])) OR ("stupor"[MeSH Terms] OR "stupor"[All Fields] OR "narcosis"[All Fields])) OR "stupor"[MeSH Terms]) AND (((((("ventilation"[MeSH Terms] OR "ventilation"[All Fields] OR "respiration"[MeSH Terms] OR "respiration"[All Fields]) OR ("ventilation"[MeSH Terms] OR "respiration"[MeSH Terms])) OR ("ventilators, mechanical"[MeSH Terms] OR ("ventilators"[All Fields] AND "mechanical"[All Fields]) OR "mechanical ventilators"[All Fields] OR "ventilator"[All Fields])) OR "ventilators, mechanical"[MeSH Terms]) OR ("oxygen"[MeSH Terms] OR "oxygen"[All Fields])) OR "oxygen"[MeSH Terms])) AND ((((("weightlessness"[MeSH Terms] OR "weightlessness"[All Fields] OR "microgravity"[All Fields]) OR "weightlessness"[MeSH Terms]) OR ("weightlessness"[MeSH Terms] OR "weightlessness"[All Fields])) OR "weightlessness"[MeSH Terms]) OR "space flight"[MeSH Terms]) | 99 |
| 44 | Does a patient in CPR in microgravity benefit from a ventilation with 100% oxygen regarding rate of ROSC compared to ventilation with room air? | ((((((((((("cardiopulmonary resuscitation"[MeSH Terms] OR ("cardiopulmonary"[All Fields] AND "resuscitation"[All Fields]) OR "cardiopulmonary resuscitation"[All Fields] OR "cpr"[All Fields]) OR "cardiopulmonary resuscitation"[MeSH Terms]) OR cardiopulmonary[All Fields]) OR "cardiopulmonary resuscitation"[MeSH Terms]) OR ("resuscitation"[MeSH Terms] OR "resuscitation"[All Fields])) OR "cardiopulmonary resuscitation"[MeSH Terms]) OR "anesthesia"[MeSH Terms]) OR ("anaesthesia"[All Fields] OR "anesthesia"[MeSH Terms] OR "anesthesia"[All Fields])) OR ("stupor"[MeSH Terms] OR "stupor"[All Fields] OR "narcosis"[All Fields])) OR "stupor"[MeSH Terms]) AND (((((("ventilation"[MeSH Terms] OR "ventilation"[All Fields] OR "respiration"[MeSH Terms] OR "respiration"[All Fields]) OR ("ventilation"[MeSH Terms] OR "respiration"[MeSH Terms])) OR ("ventilators, mechanical"[MeSH Terms] OR ("ventilators"[All Fields] AND "mechanical"[All Fields]) OR "mechanical ventilators"[All Fields] OR "ventilator"[All Fields])) OR "ventilators, mechanical"[MeSH Terms]) OR ("oxygen"[MeSH Terms] OR "oxygen"[All Fields])) OR "oxygen"[MeSH Terms])) AND ((((("weightlessness"[MeSH Terms] OR "weightlessness"[All Fields] OR "microgravity"[All Fields]) OR "weightlessness"[MeSH Terms]) OR ("weightlessness"[MeSH Terms] OR "weightlessness"[All Fields])) OR "weightlessness"[MeSH Terms]) OR "space flight"[MeSH Terms]) | 99 |
| 45 | Should the expiratory air in a patient with cardiac arrest in microgravity who is ventilated with 100% oxygen be expelled from the space vehicle or should it be kept inside regarding the risk of fire development? | ((((((((((("cardiopulmonary resuscitation"[MeSH Terms] OR ("cardiopulmonary"[All Fields] AND "resuscitation"[All Fields]) OR "cardiopulmonary resuscitation"[All Fields] OR "cpr"[All Fields]) OR "cardiopulmonary resuscitation"[MeSH Terms]) OR cardiopulmonary[All Fields]) OR "cardiopulmonary resuscitation"[MeSH Terms]) OR ("resuscitation"[MeSH Terms] OR "resuscitation"[All Fields])) OR "cardiopulmonary resuscitation"[MeSH Terms]) OR "anesthesia"[MeSH Terms]) OR ("anaesthesia"[All Fields] OR "anesthesia"[MeSH Terms] OR "anesthesia"[All Fields])) OR ("stupor"[MeSH Terms] OR "stupor"[All Fields] OR "narcosis"[All Fields])) OR "stupor"[MeSH Terms]) AND (((((("ventilation"[MeSH Terms] OR "ventilation"[All Fields] OR "respiration"[MeSH Terms] OR "respiration"[All Fields]) OR ("ventilation"[MeSH Terms] OR "respiration"[MeSH Terms])) OR ("ventilators, mechanical"[MeSH Terms] OR ("ventilators"[All Fields] AND "mechanical"[All Fields]) OR "mechanical ventilators"[All Fields] OR "ventilator"[All Fields])) OR "ventilators, mechanical"[MeSH Terms]) OR ("oxygen"[MeSH Terms] OR "oxygen"[All Fields])) OR "oxygen"[MeSH Terms])) AND ((((("weightlessness"[MeSH Terms] OR "weightlessness"[All Fields] OR "microgravity"[All Fields]) OR "weightlessness"[MeSH Terms]) OR ("weightlessness"[MeSH Terms] OR "weightlessness"[All Fields])) OR "weightlessness"[MeSH Terms]) OR "space flight"[MeSH Terms]) | 99 |
| 46 | Should capnometry be used during ventilation of a patient in cardiac arrest in microgravity regarding verification of correct airway establishment? | ((("capnography"[MeSH Terms] OR "capnography"[All Fields]) OR "capnography"[MeSH Terms]) OR "capnometry"[All Fields]) AND ((((("weightlessness"[MeSH Terms] OR "weightlessness"[All Fields] OR "microgravity"[All Fields]) OR "weightlessness"[MeSH Terms]) OR ("weightlessness"[MeSH Terms] OR "weightlessness"[All Fields])) OR "weightlessness"[MeSH Terms]) OR "space flight"[MeSH Terms]) | 0 |
| 47 | Should capnometry be used during ventilation of a patient in cardiac arrest in microgravity for quality control of CPR compared to no capnometry? | ((("capnography"[MeSH Terms] OR "capnography"[All Fields]) OR "capnography"[MeSH Terms]) OR "capnometry"[All Fields]) AND ((((("weightlessness"[MeSH Terms] OR "weightlessness"[All Fields] OR "microgravity"[All Fields]) OR "weightlessness"[MeSH Terms]) OR ("weightlessness"[MeSH Terms] OR "weightlessness"[All Fields])) OR "weightlessness"[MeSH Terms]) OR "space flight"[MeSH Terms]) | 0 |
| 48 | Should capnometry be used during ventilation of a patient in cardiac arrest in microgravity regarding the recognition of ROSC compared to no capnometry? | ((("capnography"[MeSH Terms] OR "capnography"[All Fields]) OR "capnography"[MeSH Terms]) OR "capnometry"[All Fields]) AND ((((("weightlessness"[MeSH Terms] OR "weightlessness"[All Fields] OR "microgravity"[All Fields]) OR "weightlessness"[MeSH Terms]) OR ("weightlessness"[MeSH Terms] OR "weightlessness"[All Fields])) OR "weightlessness"[MeSH Terms]) OR "space flight"[MeSH Terms]) | 0 |
| 49 | Should a quantitative capnometer be used over a simple qualitative capnometer in a patient in cardiac arrest in microgravity to verify correct ventilation and tube location? | ((("capnography"[MeSH Terms] OR "capnography"[All Fields]) OR "capnography"[MeSH Terms]) OR "capnometry"[All Fields]) AND ((((("weightlessness"[MeSH Terms] OR "weightlessness"[All Fields] OR "microgravity"[All Fields]) OR "weightlessness"[MeSH Terms]) OR ("weightlessness"[MeSH Terms] OR "weightlessness"[All Fields])) OR "weightlessness"[MeSH Terms]) OR "space flight"[MeSH Terms]) | 0 |
| 50 | Which levels of positive pressure should be used in microgravity ? | ((((((((((("cardiopulmonary resuscitation"[MeSH Terms] OR ("cardiopulmonary"[All Fields] AND "resuscitation"[All Fields]) OR "cardiopulmonary resuscitation"[All Fields] OR "cpr"[All Fields]) OR "cardiopulmonary resuscitation"[MeSH Terms]) OR cardiopulmonary[All Fields]) OR "cardiopulmonary resuscitation"[MeSH Terms]) OR ("resuscitation"[MeSH Terms] OR "resuscitation"[All Fields])) OR "cardiopulmonary resuscitation"[MeSH Terms]) OR "anesthesia"[MeSH Terms]) OR ("anaesthesia"[All Fields] OR "anesthesia"[MeSH Terms] OR "anesthesia"[All Fields])) OR ("stupor"[MeSH Terms] OR "stupor"[All Fields] OR "narcosis"[All Fields])) OR "stupor"[MeSH Terms]) AND (((((("ventilation"[MeSH Terms] OR "ventilation"[All Fields] OR "respiration"[MeSH Terms] OR "respiration"[All Fields]) OR ("ventilation"[MeSH Terms] OR "respiration"[MeSH Terms])) OR ("ventilators, mechanical"[MeSH Terms] OR ("ventilators"[All Fields] AND "mechanical"[All Fields]) OR "mechanical ventilators"[All Fields] OR "ventilator"[All Fields])) OR "ventilators, mechanical"[MeSH Terms]) OR ("oxygen"[MeSH Terms] OR "oxygen"[All Fields])) OR "oxygen"[MeSH Terms])) AND ((((("weightlessness"[MeSH Terms] OR "weightlessness"[All Fields] OR "microgravity"[All Fields]) OR "weightlessness"[MeSH Terms]) OR ("weightlessness"[MeSH Terms] OR "weightlessness"[All Fields])) OR "weightlessness"[MeSH Terms]) OR "space flight"[MeSH Terms]) | 99 |
| 51 | Is a defibrillator with adhesive electrodes more efficient regarding detection time of shockable heart rhythms in a patient in cardiac arrest in microgravity compared to a defibrillator with hard-paddles? | (((("defibrillators"[MeSH Terms] OR "electric countershock"[MeSH Terms]) OR ("defibrillators"[MeSH Terms] OR "defibrillators"[All Fields] OR "defibrillator"[All Fields])) OR ("electric countershock"[MeSH Terms] OR ("electric"[All Fields] AND "countershock"[All Fields]) OR "electric countershock"[All Fields] OR "defibrillation"[All Fields])) AND ((((("weightlessness"[MeSH Terms] OR "weightlessness"[All Fields] OR "microgravity"[All Fields]) OR "weightlessness"[MeSH Terms]) OR ("weightlessness"[MeSH Terms] OR "weightlessness"[All Fields])) OR "weightlessness"[MeSH Terms]) OR "space flight"[MeSH Terms])) AND (((((("cardiopulmonary resuscitation"[MeSH Terms] OR ("cardiopulmonary"[All Fields] AND "resuscitation"[All Fields]) OR "cardiopulmonary resuscitation"[All Fields] OR "cpr"[All Fields]) OR "cardiopulmonary resuscitation"[MeSH Terms]) OR ("cardiopulmonary resuscitation"[MeSH Terms] OR ("cardiopulmonary"[All Fields] AND "resuscitation"[All Fields]) OR "cardiopulmonary resuscitation"[All Fields])) OR "cardiopulmonary resuscitation"[MeSH Terms]) OR ("resuscitation"[MeSH Terms] OR "resuscitation"[All Fields])) OR "resuscitation"[MeSH Terms]) | 0 |
| 52 | Is a defibrillator with adhesive electrodes more efficient regarding application time in a patient in cardiac arrest in microgravity compared to a defibrillator with hard-paddles? | (((("defibrillators"[MeSH Terms] OR "electric countershock"[MeSH Terms]) OR ("defibrillators"[MeSH Terms] OR "defibrillators"[All Fields] OR "defibrillator"[All Fields])) OR ("electric countershock"[MeSH Terms] OR ("electric"[All Fields] AND "countershock"[All Fields]) OR "electric countershock"[All Fields] OR "defibrillation"[All Fields])) AND ((((("weightlessness"[MeSH Terms] OR "weightlessness"[All Fields] OR "microgravity"[All Fields]) OR "weightlessness"[MeSH Terms]) OR ("weightlessness"[MeSH Terms] OR "weightlessness"[All Fields])) OR "weightlessness"[MeSH Terms]) OR "space flight"[MeSH Terms])) AND (((((("cardiopulmonary resuscitation"[MeSH Terms] OR ("cardiopulmonary"[All Fields] AND "resuscitation"[All Fields]) OR "cardiopulmonary resuscitation"[All Fields] OR "cpr"[All Fields]) OR "cardiopulmonary resuscitation"[MeSH Terms]) OR ("cardiopulmonary resuscitation"[MeSH Terms] OR ("cardiopulmonary"[All Fields] AND "resuscitation"[All Fields]) OR "cardiopulmonary resuscitation"[All Fields])) OR "cardiopulmonary resuscitation"[MeSH Terms]) OR ("resuscitation"[MeSH Terms] OR "resuscitation"[All Fields])) OR "resuscitation"[MeSH Terms]) | 0 |
| 53 | Is a defibrillator with adhesive electrodes safer for crew and patient in a patient in cardiac arrest in microgravity compared to a defibrillator with hard-paddles? | (((("defibrillators"[MeSH Terms] OR "electric countershock"[MeSH Terms]) OR ("defibrillators"[MeSH Terms] OR "defibrillators"[All Fields] OR "defibrillator"[All Fields])) OR ("electric countershock"[MeSH Terms] OR ("electric"[All Fields] AND "countershock"[All Fields]) OR "electric countershock"[All Fields] OR "defibrillation"[All Fields])) AND ((((("weightlessness"[MeSH Terms] OR "weightlessness"[All Fields] OR "microgravity"[All Fields]) OR "weightlessness"[MeSH Terms]) OR ("weightlessness"[MeSH Terms] OR "weightlessness"[All Fields])) OR "weightlessness"[MeSH Terms]) OR "space flight"[MeSH Terms])) AND (((((("cardiopulmonary resuscitation"[MeSH Terms] OR ("cardiopulmonary"[All Fields] AND "resuscitation"[All Fields]) OR "cardiopulmonary resuscitation"[All Fields] OR "cpr"[All Fields]) OR "cardiopulmonary resuscitation"[MeSH Terms]) OR ("cardiopulmonary resuscitation"[MeSH Terms] OR ("cardiopulmonary"[All Fields] AND "resuscitation"[All Fields]) OR "cardiopulmonary resuscitation"[All Fields])) OR "cardiopulmonary resuscitation"[MeSH Terms]) OR ("resuscitation"[MeSH Terms] OR "resuscitation"[All Fields])) OR "resuscitation"[MeSH Terms]) | 0 |
| 54 | Should a patient in cardiac arrest in microgravity be restrained on an electrically isolated surface for defibrillation regarding potential electric conduction of the interior or space vehicle construction compared to no precaution? | ((((defibrillator) OR defibrillator[MeSH Terms]) OR electric defibrillation[MeSH Terms])) AND (((((weightlessness) OR weightlessness[MeSH Terms]) OR microgravity) OR microgravity[MeSH Terms]) OR space flight[MeSH Terms]) | 1 |
| 55 | Should a doctor on board of a space mission have access to a fully equipped emergency monitor/defibrillator (12-lead-EKG, SpO², RR, CO², defibrillation, pacer) when confronted with a patient in cardiac arrest in microgravity or only to an AED? | (((((("critical care"[MeSH Terms] OR ("critical"[All Fields] AND "care"[All Fields]) OR "critical care"[All Fields]) OR "critical care"[MeSH Terms]) OR ("emergencies"[MeSH Terms] OR "emergencies"[All Fields] OR "emergency"[All Fields])) OR ("emergency treatment"[MeSH Terms] OR "emergency medical services"[MeSH Terms])) OR "emergency medicine"[MeSH Terms]) AND ("monitoring, physiologic"[MeSH Terms] OR ("monitoring, physiologic"[MeSH Terms] OR ("monitoring"[All Fields] AND "physiologic"[All Fields]) OR "physiologic monitoring"[All Fields] OR "monitor"[All Fields]))) AND ((((("weightlessness"[MeSH Terms] OR "weightlessness"[All Fields]) OR "weightlessness"[MeSH Terms]) OR ("weightlessness"[MeSH Terms] OR "weightlessness"[All Fields] OR "microgravity"[All Fields])) OR "weightlessness"[MeSH Terms]) OR "space flight"[MeSH Terms]) | 21 |
| 56 | Should a crew medical officer on board of a space mission have access to a fully equipped emergency monitor/defibrillator (12-lead-EKG, SpO², RR, CO², defibrillation, pacer) when confronted with a patient in cardiac arrest in microgravity or only with an AED? | (((((("critical care"[MeSH Terms] OR ("critical"[All Fields] AND "care"[All Fields]) OR "critical care"[All Fields]) OR "critical care"[MeSH Terms]) OR ("emergencies"[MeSH Terms] OR "emergencies"[All Fields] OR "emergency"[All Fields])) OR ("emergency treatment"[MeSH Terms] OR "emergency medical services"[MeSH Terms])) OR "emergency medicine"[MeSH Terms]) AND ("monitoring, physiologic"[MeSH Terms] OR ("monitoring, physiologic"[MeSH Terms] OR ("monitoring"[All Fields] AND "physiologic"[All Fields]) OR "physiologic monitoring"[All Fields] OR "monitor"[All Fields]))) AND ((((("weightlessness"[MeSH Terms] OR "weightlessness"[All Fields]) OR "weightlessness"[MeSH Terms]) OR ("weightlessness"[MeSH Terms] OR "weightlessness"[All Fields] OR "microgravity"[All Fields])) OR "weightlessness"[MeSH Terms]) OR "space flight"[MeSH Terms]) | 21 |
| 57 | Should a normal crew member on board of a space mission have access to a fully equipped emergency monitor/defibrillator (12-lead-EKG, SpO², RR, CO², defibrillation, pacer) when confronted with a patient in cardiac arrest in microgravity or only with an AED? | (((((("critical care"[MeSH Terms] OR ("critical"[All Fields] AND "care"[All Fields]) OR "critical care"[All Fields]) OR "critical care"[MeSH Terms]) OR ("emergencies"[MeSH Terms] OR "emergencies"[All Fields] OR "emergency"[All Fields])) OR ("emergency treatment"[MeSH Terms] OR "emergency medical services"[MeSH Terms])) OR "emergency medicine"[MeSH Terms]) AND ("monitoring, physiologic"[MeSH Terms] OR ("monitoring, physiologic"[MeSH Terms] OR ("monitoring"[All Fields] AND "physiologic"[All Fields]) OR "physiologic monitoring"[All Fields] OR "monitor"[All Fields]))) AND ((((("weightlessness"[MeSH Terms] OR "weightlessness"[All Fields]) OR "weightlessness"[MeSH Terms]) OR ("weightlessness"[MeSH Terms] OR "weightlessness"[All Fields] OR "microgravity"[All Fields])) OR "weightlessness"[MeSH Terms]) OR "space flight"[MeSH Terms]) | 12 |
| 58 | Should the AED be equipped with acoustic instructions for use by crewmembers (no CMO/doctor) in a patient in cardiac arrest in microgravity compared to no instructions regarding correct electrode placement/correct chest compression depth/ correct compression&ventiltation-ratio? | ((("defibrillators"[MeSH Terms] OR "defibrillators"[All Fields] OR "defibrillator"[All Fields]) OR "defibrillators"[MeSH Terms]) OR aed[All Fields]) AND ((((("weightlessness"[MeSH Terms] OR "weightlessness"[All Fields]) OR "weightlessness"[MeSH Terms]) OR ("weightlessness"[MeSH Terms] OR "weightlessness"[All Fields] OR "microgravity"[All Fields])) OR "weightlessness"[MeSH Terms]) OR "space flight"[MeSH Terms]) | 3 |
| 59 | Should the AED be equipped with a metronome for use by crewmembers (no CMO/doctor) in a patient in cardiac arrest in microgravity compared to no metronome regarding correct electrode placement/correct chest compression depth/ correct compression&ventiltation-ratio? | ((((("weightlessness"[MeSH Terms] OR "weightlessness"[All Fields] OR "microgravity"[All Fields]) OR "weightlessness"[MeSH Terms]) OR ("weightlessness"[MeSH Terms] OR "weightlessness"[All Fields])) OR "weightlessness"[MeSH Terms]) OR "space flight"[MeSH Terms]) AND (((((("cardiopulmonary resuscitation"[MeSH Terms] OR ("cardiopulmonary"[All Fields] AND "resuscitation"[All Fields]) OR "cardiopulmonary resuscitation"[All Fields] OR "cpr"[All Fields]) OR "cardiopulmonary resuscitation"[MeSH Terms]) OR ("cardiopulmonary resuscitation"[MeSH Terms] OR ("cardiopulmonary"[All Fields] AND "resuscitation"[All Fields]) OR "cardiopulmonary resuscitation"[All Fields])) OR "cardiopulmonary resuscitation"[MeSH Terms]) OR ("resuscitation"[MeSH Terms] OR "resuscitation"[All Fields])) OR "resuscitation"[MeSH Terms]) | 34 |
| 60 | Should the AED be equipped with a feedback mechanism for use by crewmembers (no CMO/doctor) in a patient in cardiac arrest in microgravity compared to no feedback mechanism regarding correct electrode placement/correct chest compression depth/ correct compression&ventiltation-ratio? | ((((("weightlessness"[MeSH Terms] OR "weightlessness"[All Fields] OR "microgravity"[All Fields]) OR "weightlessness"[MeSH Terms]) OR ("weightlessness"[MeSH Terms] OR "weightlessness"[All Fields])) OR "weightlessness"[MeSH Terms]) OR "space flight"[MeSH Terms]) AND (((((("cardiopulmonary resuscitation"[MeSH Terms] OR ("cardiopulmonary"[All Fields] AND "resuscitation"[All Fields]) OR "cardiopulmonary resuscitation"[All Fields] OR "cpr"[All Fields]) OR "cardiopulmonary resuscitation"[MeSH Terms]) OR ("cardiopulmonary resuscitation"[MeSH Terms] OR ("cardiopulmonary"[All Fields] AND "resuscitation"[All Fields]) OR "cardiopulmonary resuscitation"[All Fields])) OR "cardiopulmonary resuscitation"[MeSH Terms]) OR ("resuscitation"[MeSH Terms] OR "resuscitation"[All Fields])) OR "resuscitation"[MeSH Terms]) | 34 |
| 61 | Is there justification for cpr in microgravity in the absence of a defib? | ((((("weightlessness"[MeSH Terms] OR "weightlessness"[All Fields] OR "microgravity"[All Fields]) OR "weightlessness"[MeSH Terms]) OR ("weightlessness"[MeSH Terms] OR "weightlessness"[All Fields])) OR "weightlessness"[MeSH Terms]) OR "space flight"[MeSH Terms]) AND (((((("cardiopulmonary resuscitation"[MeSH Terms] OR ("cardiopulmonary"[All Fields] AND "resuscitation"[All Fields]) OR "cardiopulmonary resuscitation"[All Fields] OR "cpr"[All Fields]) OR "cardiopulmonary resuscitation"[MeSH Terms]) OR ("cardiopulmonary resuscitation"[MeSH Terms] OR ("cardiopulmonary"[All Fields] AND "resuscitation"[All Fields]) OR "cardiopulmonary resuscitation"[All Fields])) OR "cardiopulmonary resuscitation"[MeSH Terms]) OR ("resuscitation"[MeSH Terms] OR "resuscitation"[All Fields])) OR "resuscitation"[MeSH Terms]) | 34 |
| 62 | Is there justification for defib in microgravity in the absence of medical skills / equipment for on-going medical support in the event of return of spontaneous circulation? | ((((("weightlessness"[MeSH Terms] OR "weightlessness"[All Fields] OR "microgravity"[All Fields]) OR "weightlessness"[MeSH Terms]) OR ("weightlessness"[MeSH Terms] OR "weightlessness"[All Fields])) OR "weightlessness"[MeSH Terms]) OR "space flight"[MeSH Terms]) AND (((((("cardiopulmonary resuscitation"[MeSH Terms] OR ("cardiopulmonary"[All Fields] AND "resuscitation"[All Fields]) OR "cardiopulmonary resuscitation"[All Fields] OR "cpr"[All Fields]) OR "cardiopulmonary resuscitation"[MeSH Terms]) OR ("cardiopulmonary resuscitation"[MeSH Terms] OR ("cardiopulmonary"[All Fields] AND "resuscitation"[All Fields]) OR "cardiopulmonary resuscitation"[All Fields])) OR "cardiopulmonary resuscitation"[MeSH Terms]) OR ("resuscitation"[MeSH Terms] OR "resuscitation"[All Fields])) OR "resuscitation"[MeSH Terms]) | 34 |
| 63 | Should reserve defibrillators/batteries be carried on the spacecraft? What risks could arise if shelf life is exceeded? | ((("exhalation"[MeSH Terms] OR "exhalation"[All Fields] OR "expiration"[All Fields]) AND date[All Fields]) OR durability[All Fields]) AND (("defibrillators"[MeSH Terms] OR "defibrillators"[All Fields] OR "defibrillator"[All Fields]) OR "defibrillators"[MeSH Terms]) | 25 |
| 64 | How many spare defibrillation electrodes should be carried on a mission (LEO vs. long-duration space mission?)? What risks could arise if shelf life is exceeded? | ((("exhalation"[MeSH Terms] OR "exhalation"[All Fields] OR "expiration"[All Fields]) AND date[All Fields]) OR durability[All Fields]) AND (("defibrillators"[MeSH Terms] OR "defibrillators"[All Fields] OR "defibrillator"[All Fields]) OR "defibrillators"[MeSH Terms]) | 25 |
| 65 | Do you need defibrillator with capability to do synchronized shock in event of supraventricular/ventricular tachycardia? | ("tachycardia, supraventricular"[MeSH Terms] OR ("tachycardia"[MeSH Terms] OR "tachycardia"[All Fields])) AND ((((("weightlessness"[MeSH Terms] OR "weightlessness"[All Fields]) OR "weightlessness"[MeSH Terms]) OR ("weightlessness"[MeSH Terms] OR "weightlessness"[All Fields] OR "microgravity"[All Fields])) OR "weightlessness"[MeSH Terms]) OR "space flight"[MeSH Terms]) | 54 |
| 66 | What is the minimum safety distance between space team and the defibrillator during the electrical shock in microgravity? | ((((defibrillator) OR defibrillator[MeSH Terms]) OR electric defibrillation[MeSH Terms])) AND (((((weightlessness) OR weightlessness[MeSH Terms]) OR microgravity) OR microgravity[MeSH Terms]) OR space flight[MeSH Terms]) | 1 |
| 67 | Should the Defibrillator be tested to work when subjected to zero gravity? | ((((defibrillator) OR defibrillator[MeSH Terms]) OR electric defibrillation[MeSH Terms])) AND (((((weightlessness) OR weightlessness[MeSH Terms]) OR microgravity) OR microgravity[MeSH Terms]) OR space flight[MeSH Terms]) | 1 |
| 68 | Is it possible to have an electromagnetic interference during shock? How would this influence safety onboard of the spacecraft? | "electromagnetic fields"[MeSH Terms] AND ((((("weightlessness"[MeSH Terms] OR "weightlessness"[All Fields]) OR "weightlessness"[MeSH Terms]) OR ("weightlessness"[MeSH Terms] OR "weightlessness"[All Fields] OR "microgravity"[All Fields])) OR "weightlessness"[MeSH Terms]) OR "space flight"[MeSH Terms]) | 32 |
| 69 | Can the application of an intravenous catheter at the arm be performed quicker than the insertion of an intraosseous needle at the tibial tuberosity (EZ-IO) in a patient in cardiac arrest in microgravity? | (((("injections, intravenous"[MeSH Terms] OR intravenous[All Fields]) OR "infusions, intravenous"[MeSH Terms]) OR "infusions, intraosseous"[MeSH Terms]) OR intraosseous[All Fields]) AND ((((("weightlessness"[MeSH Terms] OR "weightlessness"[All Fields]) OR "weightlessness"[MeSH Terms]) OR ("weightlessness"[MeSH Terms] OR "weightlessness"[All Fields] OR "microgravity"[All Fields])) OR "weightlessness"[MeSH Terms]) OR "space flight"[MeSH Terms]) | 47 |
| 70 | Is the success rate of an intravenous catheter application at the arm higher than the insertion of an intraosseous needle at the tibial tuberosity(EZ-IO) in a patient in cardiac arrest in microgravity? | (((("injections, intravenous"[MeSH Terms] OR intravenous[All Fields]) OR "infusions, intravenous"[MeSH Terms]) OR "infusions, intraosseous"[MeSH Terms]) OR intraosseous[All Fields]) AND ((((("weightlessness"[MeSH Terms] OR "weightlessness"[All Fields]) OR "weightlessness"[MeSH Terms]) OR ("weightlessness"[MeSH Terms] OR "weightlessness"[All Fields] OR "microgravity"[All Fields])) OR "weightlessness"[MeSH Terms]) OR "space flight"[MeSH Terms]) | 47 |
| 71 | Is a drilling machine-type intraosseous device more effective regarding successful insertion rate than a spring-type intraosseous device in a patient in cardiac arrest in microgravity? | ("infusions, intraosseous"[MeSH Terms] OR intraosseous[All Fields]) AND ((((("weightlessness"[MeSH Terms] OR "weightlessness"[All Fields]) OR "weightlessness"[MeSH Terms]) OR ("weightlessness"[MeSH Terms] OR "weightlessness"[All Fields] OR "microgravity"[All Fields])) OR "weightlessness"[MeSH Terms]) OR "space flight"[MeSH Terms]) | 2 |
| 72 | Is a drilling machine-type intraosseous device more effective regarding insertion time than a spring-type intraosseous device in a patient in cardiac arrest in microgravity? | ("infusions, intraosseous"[MeSH Terms] OR intraosseous[All Fields]) AND ((((("weightlessness"[MeSH Terms] OR "weightlessness"[All Fields]) OR "weightlessness"[MeSH Terms]) OR ("weightlessness"[MeSH Terms] OR "weightlessness"[All Fields] OR "microgravity"[All Fields])) OR "weightlessness"[MeSH Terms]) OR "space flight"[MeSH Terms]) | 2 |
| 73 | Is a drilling machine-type intraosseous device safer regarding complication rate than a spring-type intraosseous device in a patient in cardiac arrest in microgravity? | ("infusions, intraosseous"[MeSH Terms] OR intraosseous[All Fields]) AND ((((("weightlessness"[MeSH Terms] OR "weightlessness"[All Fields]) OR "weightlessness"[MeSH Terms]) OR ("weightlessness"[MeSH Terms] OR "weightlessness"[All Fields] OR "microgravity"[All Fields])) OR "weightlessness"[MeSH Terms]) OR "space flight"[MeSH Terms]) | 2 |
| 74 | Is a drilling machine-type intraosseous device safer regarding possible damage to the space vehicle than a spring-type intraosseous device in a patient in cardiac arrest in microgravity? | ("infusions, intraosseous"[MeSH Terms] OR intraosseous[All Fields]) AND ((((("weightlessness"[MeSH Terms] OR "weightlessness"[All Fields]) OR "weightlessness"[MeSH Terms]) OR ("weightlessness"[MeSH Terms] OR "weightlessness"[All Fields] OR "microgravity"[All Fields])) OR "weightlessness"[MeSH Terms]) OR "space flight"[MeSH Terms]) | 2 |
| 75 | Is a drilling machine-type intraosseous device more effective regarding successful insertion rate than a hand-driven needle in a patient in cardiac arrest in microgravity? | ("infusions, intraosseous"[MeSH Terms] OR intraosseous[All Fields]) AND ((((("weightlessness"[MeSH Terms] OR "weightlessness"[All Fields]) OR "weightlessness"[MeSH Terms]) OR ("weightlessness"[MeSH Terms] OR "weightlessness"[All Fields] OR "microgravity"[All Fields])) OR "weightlessness"[MeSH Terms]) OR "space flight"[MeSH Terms]) | 2 |
| 76 | Is a drilling machine-type intraosseous device more effective regarding insertion time than a hand-driven needle in a patient in cardiac arrest in microgravity? | ("infusions, intraosseous"[MeSH Terms] OR intraosseous[All Fields]) AND ((((("weightlessness"[MeSH Terms] OR "weightlessness"[All Fields]) OR "weightlessness"[MeSH Terms]) OR ("weightlessness"[MeSH Terms] OR "weightlessness"[All Fields] OR "microgravity"[All Fields])) OR "weightlessness"[MeSH Terms]) OR "space flight"[MeSH Terms]) | 2 |
| 77 | Is a drilling machine-type intraosseous device safer regarding complication rate than a hand-driven needle in a patient in cardiac arrest in microgravity? | ("infusions, intraosseous"[MeSH Terms] OR intraosseous[All Fields]) AND ((((("weightlessness"[MeSH Terms] OR "weightlessness"[All Fields]) OR "weightlessness"[MeSH Terms]) OR ("weightlessness"[MeSH Terms] OR "weightlessness"[All Fields] OR "microgravity"[All Fields])) OR "weightlessness"[MeSH Terms]) OR "space flight"[MeSH Terms]) | 2 |
| 78 | Is a drilling machine-type intraosseous device safer regarding possible damage to the space vehicle than a hand-driven needle in a patient in cardiac arrest in microgravity? | ("infusions, intraosseous"[MeSH Terms] OR intraosseous[All Fields]) AND ((((("weightlessness"[MeSH Terms] OR "weightlessness"[All Fields]) OR "weightlessness"[MeSH Terms]) OR ("weightlessness"[MeSH Terms] OR "weightlessness"[All Fields] OR "microgravity"[All Fields])) OR "weightlessness"[MeSH Terms]) OR "space flight"[MeSH Terms]) | 2 |
| 79 | Is the use of standard CPR medication (1mg/10ml epinephrine, 300mg amiodarone) in ready-to-use plastic syringes superior compared to normal glass ampoules regarding application time in a patient in cardiac arrest in microgravity? | (((((((medication) OR medication[MeSH Terms]) OR intravenous injection[MeSH Terms]) OR intravenous) OR syringe[MeSH Terms]) OR syringe)) AND (((((weightlessness) OR weightlessness[MeSH Terms]) OR microgravity) OR microgravity[MeSH Terms]) OR space flight[MeSH Terms]) | 368 |
| 80 | Is the use of standard CPR medication (1mg/10ml epinephrine, 300mg amiodarone) in ready-to-use plastic syringes safer compared to normal glass ampoules regarding environment contamination with package parts (e.g. glass splinters) in a patient in cardiac arrest in microgravity? | (((((((medication) OR medication[MeSH Terms]) OR intravenous injection[MeSH Terms]) OR intravenous) OR syringe[MeSH Terms]) OR syringe)) AND (((((weightlessness) OR weightlessness[MeSH Terms]) OR microgravity) OR microgravity[MeSH Terms]) OR space flight[MeSH Terms]) | 368 |
| 81 | Is the use of standard CPR medication (1mg/10ml epinephrine, 300mg amiodarone) in plastic ampoules superior compared to normal glass ampoules regarding application time in a patient in cardiac arrest in microgravity? | (((((((medication) OR medication[MeSH Terms]) OR intravenous injection[MeSH Terms]) OR intravenous) OR syringe[MeSH Terms]) OR syringe)) AND (((((weightlessness) OR weightlessness[MeSH Terms]) OR microgravity) OR microgravity[MeSH Terms]) OR space flight[MeSH Terms]) | 368 |
| 82 | Is the use of standard CPR medication (1mg/10ml epinephrine, 300mg amiodarone) in plastic ampoules safer compared to normal glass ampoules regarding environment contamination with package parts (e.g. glass splinters) in a patient in cardiac arrest in microgravity? | (((((((medication) OR medication[MeSH Terms]) OR intravenous injection[MeSH Terms]) OR intravenous) OR syringe[MeSH Terms]) OR syringe)) AND (((((weightlessness) OR weightlessness[MeSH Terms]) OR microgravity) OR microgravity[MeSH Terms]) OR space flight[MeSH Terms]) | 368 |
| 83 | Is it reasonable to accept the higher transport volume of ready-to-use plastic syringes compared to the standard material for glass ampoules in spaceflight missions? | (((((((medication) OR medication[MeSH Terms]) OR intravenous injection[MeSH Terms]) OR intravenous) OR syringe[MeSH Terms]) OR syringe)) AND (((((weightlessness) OR weightlessness[MeSH Terms]) OR microgravity) OR microgravity[MeSH Terms]) OR space flight[MeSH Terms]) | 368 |
| 84 | Is the application of intravenous fluids via syringe pump more effective regarding consistency of infusion rate than the use of an infusion bag fixed into a blood pressure cuff in a patient in cardiac arrest in microgravity? | ((("syringes"[MeSH Terms] OR "syringes"[All Fields] OR "syringe"[All Fields]) AND pump[All Fields]) OR (("pressure"[MeSH Terms] OR "pressure"[All Fields]) AND infusion[All Fields])) AND ((((("weightlessness"[MeSH Terms] OR "weightlessness"[All Fields]) OR "weightlessness"[MeSH Terms]) OR ("weightlessness"[MeSH Terms] OR "weightlessness"[All Fields] OR "microgravity"[All Fields])) OR "weightlessness"[MeSH Terms]) OR "space flight"[MeSH Terms]) | 30 |
| 85 | Should first aid training for the whole crew include CPR training in simulated microgravity compared to training in sea level gravity? | ((("first aid"[MeSH Terms] OR ("first aid"[MeSH Terms] OR ("first"[All Fields] AND "aid"[All Fields]) OR "first aid"[All Fields])) OR ("education"[Subheading] OR "education"[All Fields] OR "training"[All Fields] OR "education"[MeSH Terms] OR "training"[All Fields])) AND ((((("weightlessness"[MeSH Terms] OR "weightlessness"[All Fields]) OR "weightlessness"[MeSH Terms]) OR ("weightlessness"[MeSH Terms] OR "weightlessness"[All Fields] OR "microgravity"[All Fields])) OR "weightlessness"[MeSH Terms]) OR "space flight"[MeSH Terms])) AND ((((("cardiopulmonary resuscitation"[MeSH Terms] OR ("cardiopulmonary"[All Fields] AND "resuscitation"[All Fields]) OR "cardiopulmonary resuscitation"[All Fields] OR "cpr"[All Fields]) OR "cardiopulmonary resuscitation"[MeSH Terms]) OR ("resuscitation"[MeSH Terms] OR "resuscitation"[All Fields])) OR "cardiopulmonary resuscitation"[MeSH Terms]) OR "resuscitation"[MeSH Terms]) | 10 |
| 86 | Should the normal crewmembers be trained in ALS compared to normal first aid/BLS training for CPR in microgravity? | ((("first aid"[MeSH Terms] OR ("first aid"[MeSH Terms] OR ("first"[All Fields] AND "aid"[All Fields]) OR "first aid"[All Fields])) OR ("education"[Subheading] OR "education"[All Fields] OR "training"[All Fields] OR "education"[MeSH Terms] OR "training"[All Fields])) AND ((((("weightlessness"[MeSH Terms] OR "weightlessness"[All Fields]) OR "weightlessness"[MeSH Terms]) OR ("weightlessness"[MeSH Terms] OR "weightlessness"[All Fields] OR "microgravity"[All Fields])) OR "weightlessness"[MeSH Terms]) OR "space flight"[MeSH Terms])) AND ((((("cardiopulmonary resuscitation"[MeSH Terms] OR ("cardiopulmonary"[All Fields] AND "resuscitation"[All Fields]) OR "cardiopulmonary resuscitation"[All Fields] OR "cpr"[All Fields]) OR "cardiopulmonary resuscitation"[MeSH Terms]) OR ("resuscitation"[MeSH Terms] OR "resuscitation"[All Fields])) OR "cardiopulmonary resuscitation"[MeSH Terms]) OR "resuscitation"[MeSH Terms]) | 10 |
| 87 | Should controlled mild hypothermia be induced in a patient with ROSC after cardiac arrest in microgravity compared to normothermia regarding neurological outcome? | ("hypothermia"[MeSH Terms] OR ("hypothermia"[MeSH Terms] OR "hypothermia"[All Fields])) AND ((((("weightlessness"[MeSH Terms] OR "weightlessness"[All Fields]) OR "weightlessness"[MeSH Terms]) OR ("weightlessness"[MeSH Terms] OR "weightlessness"[All Fields] OR "microgravity"[All Fields])) OR "weightlessness"[MeSH Terms]) OR "space flight"[MeSH Terms]) | 19 |
| 88 | Should controlled mild hypothermia be induced in a patient with ROSC after cardiac arrest in microgravity compared to normothermia regarding survival rate after (insert variable timeframe)? | ("hypothermia"[MeSH Terms] OR ("hypothermia"[MeSH Terms] OR "hypothermia"[All Fields])) AND ((((("weightlessness"[MeSH Terms] OR "weightlessness"[All Fields]) OR "weightlessness"[MeSH Terms]) OR ("weightlessness"[MeSH Terms] OR "weightlessness"[All Fields] OR "microgravity"[All Fields])) OR "weightlessness"[MeSH Terms]) OR "space flight"[MeSH Terms]) | 19 |
| 89 | Which SGA is best suited for use in microgravity? Laryngeal tube, I-gel, laryngeal mask or combitube? | ((((((airway[All Fields] OR "airway management"[MeSH Terms]) OR ("laryngeal masks"[MeSH Terms] OR ("laryngeal"[All Fields] AND "masks"[All Fields]) OR "laryngeal masks"[All Fields] OR ("laryngeal"[All Fields] AND "mask"[All Fields]) OR "laryngeal mask"[All Fields])) OR "laryngeal masks"[MeSH Terms]) OR i-gel[All Fields]) OR combitube[All Fields]) OR (("larynx"[MeSH Terms] OR "larynx"[All Fields] OR "laryngeal"[All Fields]) AND tube[All Fields])) AND ((((("weightlessness"[MeSH Terms] OR "weightlessness"[All Fields]) OR "weightlessness"[MeSH Terms]) OR ("weightlessness"[MeSH Terms] OR "weightlessness"[All Fields] OR "microgravity"[All Fields])) OR "weightlessness"[MeSH Terms]) OR "space flight"[MeSH Terms]) | 54 |
| 90 | Should an SGA with an option for later endotracheal intubation be used? I-LTSD, intubation laryngeal mask or LMA fastrach? | ((((("weightlessness"[MeSH Terms] OR "weightlessness"[All Fields] OR "microgravity"[All Fields]) OR "weightlessness"[MeSH Terms]) OR ("weightlessness"[MeSH Terms] OR "weightlessness"[All Fields])) OR "weightlessness"[MeSH Terms]) OR "space flight"[MeSH Terms]) AND ((((((((((("intubation"[MeSH Terms] OR "intubation"[All Fields]) OR "intubation"[MeSH Terms]) OR endotracheal[All Fields]) OR "intubation, intratracheal"[MeSH Terms]) OR "airway management"[MeSH Terms]) OR airway[All Fields]) OR "laryngeal masks"[MeSH Terms]) OR ("laryngeal masks"[MeSH Terms] OR ("laryngeal"[All Fields] AND "masks"[All Fields]) OR "laryngeal masks"[All Fields] OR ("laryngeal"[All Fields] AND "mask"[All Fields]) OR "laryngeal mask"[All Fields])) OR combitube[All Fields]) OR (("larynx"[MeSH Terms] OR "larynx"[All Fields] OR "laryngeal"[All Fields]) AND tube[All Fields])) OR i-gel[All Fields]) | 55 |
| 91 | Should the CMO be trained in surgical cricothyrotomy for a can-not-ventilate-can-not-intubate-situation in a patient in cardiac arrest in microgravity? | cricothyrotomy[All Fields] AND ((((("weightlessness"[MeSH Terms] OR "weightlessness"[All Fields]) OR "weightlessness"[MeSH Terms]) OR ("weightlessness"[MeSH Terms] OR "weightlessness"[All Fields] OR "microgravity"[All Fields])) OR "weightlessness"[MeSH Terms]) OR "space flight"[MeSH Terms]) | 0 |
| 92 | Do electrical suction units (like the Weinmann Accuvac Pro/ Laerdal LSU/ Laerdal CSU4) work properly in microgravity? | (("suction"[MeSH Terms] OR ("suction"[MeSH Terms] OR "suction"[All Fields])) OR aspiration[All Fields]) AND ((((("weightlessness"[MeSH Terms] OR "weightlessness"[All Fields] OR "microgravity"[All Fields]) OR "weightlessness"[MeSH Terms]) OR ("weightlessness"[MeSH Terms] OR "weightlessness"[All Fields])) OR "weightlessness"[MeSH Terms]) OR "space flight"[MeSH Terms]) | 38 |
| 93 | Do any other manual suction units except for a syringe-style suction work in microgravity? | (("suction"[MeSH Terms] OR ("suction"[MeSH Terms] OR "suction"[All Fields])) OR aspiration[All Fields]) AND ((((("weightlessness"[MeSH Terms] OR "weightlessness"[All Fields] OR "microgravity"[All Fields]) OR "weightlessness"[MeSH Terms]) OR ("weightlessness"[MeSH Terms] OR "weightlessness"[All Fields])) OR "weightlessness"[MeSH Terms]) OR "space flight"[MeSH Terms]) | 38 |
| 94 | Should an intravenous access always be attempted before insertion of an intraosseous needle in a patient in cardiac arrest in microgravity? | (((("injections, intravenous"[MeSH Terms] OR intravenous[All Fields]) OR "infusions, intravenous"[MeSH Terms]) OR "infusions, intraosseous"[MeSH Terms]) OR intraosseous[All Fields]) AND ((((("weightlessness"[MeSH Terms] OR "weightlessness"[All Fields]) OR "weightlessness"[MeSH Terms]) OR ("weightlessness"[MeSH Terms] OR "weightlessness"[All Fields] OR "microgravity"[All Fields])) OR "weightlessness"[MeSH Terms]) OR "space flight"[MeSH Terms]) | 47 |
| 95 | How do you handle medication or medical equipment with expiration dates on a space mission that exceeds those dates? | ((("pharmaceutical preparations"[MeSH Terms] OR ("pharmaceutical"[All Fields] AND "preparations"[All Fields]) OR "pharmaceutical preparations"[All Fields] OR "medication"[All Fields]) OR (("exhalation"[MeSH Terms] OR "exhalation"[All Fields] OR "expiration"[All Fields]) AND date[All Fields])) OR expiry[All Fields]) AND ((((("weightlessness"[MeSH Terms] OR "weightlessness"[All Fields]) OR "weightlessness"[MeSH Terms]) OR ("weightlessness"[MeSH Terms] OR "weightlessness"[All Fields] OR "microgravity"[All Fields])) OR "weightlessness"[MeSH Terms]) OR "space flight"[MeSH Terms]) | 315 |
| 96 | How does the effect of epinephrine change after its expiration date? Can it be conserved for more than one year? | (("epinephrine"[MeSH Terms] OR "epinephrine"[All Fields]) AND (expiry[All Fields] AND date[All Fields])) OR (("exhalation"[MeSH Terms] OR "exhalation"[All Fields] OR "expiration"[All Fields]) AND date[All Fields]) | 445 |
| 97 | How does the effect of amiodarone change after its expiration date? Can it be conserved for more than two years? | (("amiodarone"[MeSH Terms] OR "amiodarone"[All Fields]) AND (expiry[All Fields] AND date[All Fields])) OR (("exhalation"[MeSH Terms] OR "exhalation"[All Fields] OR "expiration"[All Fields]) AND date[All Fields]) | 443 |
| 98 | Is ROSC recognition different than in normal gravity? | (((rosc) OR return of spontaneous circulation)) AND (((((weightlessness) OR weightlessness[MeSH Terms]) OR microgravity) OR microgravity[MeSH Terms]) OR space flight[MeSH Terms]) | 1 |
| 99 | How should be dealt with the corpse of a crewmember after unsuccessful CPR? | ((("death"[MeSH Terms] OR "death"[All Fields]) OR ("cadaver"[MeSH Terms] OR "cadaver"[All Fields] OR "corpse"[All Fields])) OR ("cadaver"[MeSH Terms] OR "cadaver"[All Fields])) AND ((((("weightlessness"[MeSH Terms] OR "weightlessness"[All Fields]) OR "weightlessness"[MeSH Terms]) OR ("weightlessness"[MeSH Terms] OR "weightlessness"[All Fields] OR "microgravity"[All Fields])) OR "weightlessness"[MeSH Terms]) OR "space flight"[MeSH Terms]) | 166 |
| 100 | Should the corpse of a crewmember after unsuccessful CPR be stored inside the space vehicle or should it be removed? | ((("death"[MeSH Terms] OR "death"[All Fields]) OR ("cadaver"[MeSH Terms] OR "cadaver"[All Fields] OR "corpse"[All Fields])) OR ("cadaver"[MeSH Terms] OR "cadaver"[All Fields])) AND ((((("weightlessness"[MeSH Terms] OR "weightlessness"[All Fields]) OR "weightlessness"[MeSH Terms]) OR ("weightlessness"[MeSH Terms] OR "weightlessness"[All Fields] OR "microgravity"[All Fields])) OR "weightlessness"[MeSH Terms]) OR "space flight"[MeSH Terms]) | 166 |
| 101 | If the corpse is to be stored inside the space vehicle, in what kind of compartment should it be stored? Plastic body bag? Special Box? In which part of the space vehicle? | ((("death"[MeSH Terms] OR "death"[All Fields]) OR ("cadaver"[MeSH Terms] OR "cadaver"[All Fields] OR "corpse"[All Fields])) OR ("cadaver"[MeSH Terms] OR "cadaver"[All Fields])) AND ((((("weightlessness"[MeSH Terms] OR "weightlessness"[All Fields]) OR "weightlessness"[MeSH Terms]) OR ("weightlessness"[MeSH Terms] OR "weightlessness"[All Fields] OR "microgravity"[All Fields])) OR "weightlessness"[MeSH Terms]) OR "space flight"[MeSH Terms]) | 166 |
| 102 | Is it possible to burn the body or in any other way produce a reasonably small package of human remains to transport those remains back to earth? Could they be stored outside the space vehicle but enabling them to survive the reentry into earth orbit? | ((("death"[MeSH Terms] OR "death"[All Fields]) OR ("cadaver"[MeSH Terms] OR "cadaver"[All Fields] OR "corpse"[All Fields])) OR ("cadaver"[MeSH Terms] OR "cadaver"[All Fields])) AND ((((("weightlessness"[MeSH Terms] OR "weightlessness"[All Fields]) OR "weightlessness"[MeSH Terms]) OR ("weightlessness"[MeSH Terms] OR "weightlessness"[All Fields] OR "microgravity"[All Fields])) OR "weightlessness"[MeSH Terms]) OR "space flight"[MeSH Terms]) | 166 |
| 103 | If the corpse of a crewmember should not be stored inside the space vehicle in what way should he be dumped into the open space? Body bag? Coffin? | ((("death"[MeSH Terms] OR "death"[All Fields]) OR ("cadaver"[MeSH Terms] OR "cadaver"[All Fields] OR "corpse"[All Fields])) OR ("cadaver"[MeSH Terms] OR "cadaver"[All Fields])) AND ((((("weightlessness"[MeSH Terms] OR "weightlessness"[All Fields]) OR "weightlessness"[MeSH Terms]) OR ("weightlessness"[MeSH Terms] OR "weightlessness"[All Fields] OR "microgravity"[All Fields])) OR "weightlessness"[MeSH Terms]) OR "space flight"[MeSH Terms]) | 166 |
| 104 | What possible consequences arise from that decision? Possible contamination of space with biomaterial? What if a dead crewmember is dumped on or near Mars? Could future missions searching for life on Mars falsely recognize those human remains as an evidence for life on Mars? | ((("death"[MeSH Terms] OR "death"[All Fields]) OR ("cadaver"[MeSH Terms] OR "cadaver"[All Fields] OR "corpse"[All Fields])) OR ("cadaver"[MeSH Terms] OR "cadaver"[All Fields])) AND ((((("weightlessness"[MeSH Terms] OR "weightlessness"[All Fields]) OR "weightlessness"[MeSH Terms]) OR ("weightlessness"[MeSH Terms] OR "weightlessness"[All Fields] OR "microgravity"[All Fields])) OR "weightlessness"[MeSH Terms]) OR "space flight"[MeSH Terms]) | 166 |
| 105 | How can the remaining crew members deal with the death of their comrade and the decision regarding his corpse? | ((("death"[MeSH Terms] OR "death"[All Fields]) OR ("cadaver"[MeSH Terms] OR "cadaver"[All Fields] OR "corpse"[All Fields])) OR ("cadaver"[MeSH Terms] OR "cadaver"[All Fields])) AND ((((("weightlessness"[MeSH Terms] OR "weightlessness"[All Fields]) OR "weightlessness"[MeSH Terms]) OR ("weightlessness"[MeSH Terms] OR "weightlessness"[All Fields] OR "microgravity"[All Fields])) OR "weightlessness"[MeSH Terms]) OR "space flight"[MeSH Terms]) | 166 |
| 106 | Should an electrical ventilator, independent of a gas source, (like the SAVeII , AutoMedx) be used for a patient with ROSC in a space mission for prolonged ventilation regarding gas supply? | ("ventilators, mechanical"[MeSH Terms] OR ("ventilators"[All Fields] AND "mechanical"[All Fields]) OR "mechanical ventilators"[All Fields] OR "ventilator"[All Fields]) AND ((((("weightlessness"[MeSH Terms] OR "weightlessness"[All Fields] OR "microgravity"[All Fields]) OR "weightlessness"[MeSH Terms]) OR ("weightlessness"[MeSH Terms] OR "weightlessness"[All Fields])) OR "weightlessness"[MeSH Terms]) OR "space flight"[MeSH Terms]) | 9 |
| 107 | Should an electrical ventilator, independent of a gas source, (like the SAVeII , AutoMedx) be used for a patient with ROSC in a space mission for prolonged ventilation regarding safety issues (flammability if 100% oxygen is used in a gas operated ventilator)? | ("ventilators, mechanical"[MeSH Terms] OR ("ventilators"[All Fields] AND "mechanical"[All Fields]) OR "mechanical ventilators"[All Fields] OR "ventilator"[All Fields]) AND ((((("weightlessness"[MeSH Terms] OR "weightlessness"[All Fields] OR "microgravity"[All Fields]) OR "weightlessness"[MeSH Terms]) OR ("weightlessness"[MeSH Terms] OR "weightlessness"[All Fields])) OR "weightlessness"[MeSH Terms]) OR "space flight"[MeSH Terms]) | 9 |
| 108 | How can intensive care in a patient with ROSC in a space mission be guaranteed regarding medication supply (sedation, iv-fluids, catecholamines, antibiotics, nutritional fluids)? | (("critical care"[MeSH Terms] OR ("critical care"[MeSH Terms] OR ("critical"[All Fields] AND "care"[All Fields]) OR "critical care"[All Fields] OR ("intensive"[All Fields] AND "care"[All Fields]) OR "intensive care"[All Fields])) OR "intensive care units"[MeSH Terms]) AND ((((("weightlessness"[MeSH Terms] OR "weightlessness"[All Fields] OR "microgravity"[All Fields]) OR "weightlessness"[MeSH Terms]) OR ("weightlessness"[MeSH Terms] OR "weightlessness"[All Fields])) OR "weightlessness"[MeSH Terms]) OR "space flight"[MeSH Terms]) | 65 |
| 109 | How can intensive care in a patient with ROSC in a space mission be guaranteed regarding monitoring? | (("critical care"[MeSH Terms] OR ("critical care"[MeSH Terms] OR ("critical"[All Fields] AND "care"[All Fields]) OR "critical care"[All Fields] OR ("intensive"[All Fields] AND "care"[All Fields]) OR "intensive care"[All Fields])) OR "intensive care units"[MeSH Terms]) AND ((((("weightlessness"[MeSH Terms] OR "weightlessness"[All Fields] OR "microgravity"[All Fields]) OR "weightlessness"[MeSH Terms]) OR ("weightlessness"[MeSH Terms] OR "weightlessness"[All Fields])) OR "weightlessness"[MeSH Terms]) OR "space flight"[MeSH Terms]) | 65 |
| 110 | How can intensive care in a patient with ROSC in a space mission be guaranteed regarding basic care (urination/defecation/positioning of the patient/hygiene)? | (("critical care"[MeSH Terms] OR ("critical care"[MeSH Terms] OR ("critical"[All Fields] AND "care"[All Fields]) OR "critical care"[All Fields] OR ("intensive"[All Fields] AND "care"[All Fields]) OR "intensive care"[All Fields])) OR "intensive care units"[MeSH Terms]) AND ((((("weightlessness"[MeSH Terms] OR "weightlessness"[All Fields] OR "microgravity"[All Fields]) OR "weightlessness"[MeSH Terms]) OR ("weightlessness"[MeSH Terms] OR "weightlessness"[All Fields])) OR "weightlessness"[MeSH Terms]) OR "space flight"[MeSH Terms]) | 65 |
| 111 | How can weaning be accomplished in a patient with ROSC in a space mission after a prolonged phase of ventilation? | ("weaning"[MeSH Terms] OR ("weaning"[MeSH Terms] OR "weaning"[All Fields])) AND ((((("weightlessness"[MeSH Terms] OR "weightlessness"[All Fields] OR "microgravity"[All Fields]) OR "weightlessness"[MeSH Terms]) OR ("weightlessness"[MeSH Terms] OR "weightlessness"[All Fields])) OR "weightlessness"[MeSH Terms]) OR "space flight"[MeSH Terms]) | 4 |
| 112 | Can telemedicine support the crew during CPR of a patient in cardiac arrest in microgravity in low earth orbit? | (((((("critical care"[MeSH Terms] OR ("critical"[All Fields] AND "care"[All Fields]) OR "critical care"[All Fields]) OR "critical care"[MeSH Terms]) OR ("emergencies"[MeSH Terms] OR "emergencies"[All Fields] OR "emergency"[All Fields])) OR "emergency medicine"[MeSH Terms]) OR ("emergency treatment"[MeSH Terms] OR "emergency medical services"[MeSH Terms])) AND (((("telemedicine"[MeSH Terms] OR "telemedicine"[All Fields]) OR "telemedicine"[MeSH Terms]) OR ("telemetry"[MeSH Terms] OR "telemetry"[All Fields])) OR "telemetry"[MeSH Terms])) AND ((((("weightlessness"[MeSH Terms] OR "weightlessness"[All Fields] OR "microgravity"[All Fields]) OR "weightlessness"[MeSH Terms]) OR ("weightlessness"[MeSH Terms] OR "weightlessness"[All Fields])) OR "weightlessness"[MeSH Terms]) OR "space flight"[MeSH Terms]) | 25 |
| 113 | Can telemedicine support the crew during CPR of a patient in cardiac arrest in microgravity on a mars mission? What would be the expected time delay for communication from mars to earth? | (((((("critical care"[MeSH Terms] OR ("critical"[All Fields] AND "care"[All Fields]) OR "critical care"[All Fields]) OR "critical care"[MeSH Terms]) OR ("emergencies"[MeSH Terms] OR "emergencies"[All Fields] OR "emergency"[All Fields])) OR "emergency medicine"[MeSH Terms]) OR ("emergency treatment"[MeSH Terms] OR "emergency medical services"[MeSH Terms])) AND (((("telemedicine"[MeSH Terms] OR "telemedicine"[All Fields]) OR "telemedicine"[MeSH Terms]) OR ("telemetry"[MeSH Terms] OR "telemetry"[All Fields])) OR "telemetry"[MeSH Terms])) AND ((((("weightlessness"[MeSH Terms] OR "weightlessness"[All Fields] OR "microgravity"[All Fields]) OR "weightlessness"[MeSH Terms]) OR ("weightlessness"[MeSH Terms] OR "weightlessness"[All Fields])) OR "weightlessness"[MeSH Terms]) OR "space flight"[MeSH Terms]) | 25 |
| 114 | Could teleanaesthesia (E.G. automated intubation ) play a role in space CPR? | (((((teleanesthesia[All Fields] OR "anesthesia"[MeSH Terms]) OR ("anaesthesia"[All Fields] OR "anesthesia"[MeSH Terms] OR "anesthesia"[All Fields])) OR ("intubation"[MeSH Terms] OR "intubation"[All Fields])) OR "intubation"[MeSH Terms]) AND (((("telemedicine"[MeSH Terms] OR "telemedicine"[All Fields]) OR "telemedicine"[MeSH Terms]) OR ("telemetry"[MeSH Terms] OR "telemetry"[All Fields])) OR "telemetry"[MeSH Terms])) AND ((((("weightlessness"[MeSH Terms] OR "weightlessness"[All Fields] OR "microgravity"[All Fields]) OR "weightlessness"[MeSH Terms]) OR ("weightlessness"[MeSH Terms] OR "weightlessness"[All Fields])) OR "weightlessness"[MeSH Terms]) OR "space flight"[MeSH Terms]) | 5 |
| 115 | Should the medical training for CMOs be extended above the 80-hour course and include advanced techniques for ALS and treatment of reversible causes for cardiac arrest? | ((((medical[All Fields] AND ("education"[Subheading] OR "education"[All Fields] OR "training"[All Fields] OR "education"[MeSH Terms] OR "training"[All Fields])) OR (medical[All Fields] AND skills[All Fields])) OR ("education, medical"[MeSH Terms] OR ("education"[All Fields] AND "medical"[All Fields]) OR "medical education"[All Fields] OR ("medical"[All Fields] AND "education"[All Fields]))) OR (("first aid"[MeSH Terms] OR ("first"[All Fields] AND "aid"[All Fields]) OR "first aid"[All Fields]) AND ("education"[Subheading] OR "education"[All Fields] OR "training"[All Fields] OR "education"[MeSH Terms] OR "training"[All Fields]))) AND ((((("weightlessness"[MeSH Terms] OR "weightlessness"[All Fields] OR "microgravity"[All Fields]) OR "weightlessness"[MeSH Terms]) OR ("weightlessness"[MeSH Terms] OR "weightlessness"[All Fields])) OR "weightlessness"[MeSH Terms]) OR "space flight"[MeSH Terms]) | 245 |
| 116 | How should a possible Crew Medical Doctor (CMD) be trained and what medical branch should he be specialized in? | ((("physicians"[MeSH Terms] OR "physicians"[All Fields] OR "doctor"[All Fields]) OR ("physicians"[MeSH Terms] OR "physicians"[All Fields] OR "physician"[All Fields])) AND ((((("critical care"[MeSH Terms] OR ("critical"[All Fields] AND "care"[All Fields]) OR "critical care"[All Fields]) OR ("emergencies"[MeSH Terms] OR "emergencies"[All Fields] OR "emergency"[All Fields])) OR ("cardiopulmonary resuscitation"[MeSH Terms] OR ("cardiopulmonary"[All Fields] AND "resuscitation"[All Fields]) OR "cardiopulmonary resuscitation"[All Fields] OR "cpr"[All Fields])) OR cardiopulmonary[All Fields]) OR ("resuscitation"[MeSH Terms] OR "resuscitation"[All Fields]))) AND ((((("weightlessness"[MeSH Terms] OR "weightlessness"[All Fields] OR "microgravity"[All Fields]) OR "weightlessness"[MeSH Terms]) OR ("weightlessness"[MeSH Terms] OR "weightlessness"[All Fields])) OR "weightlessness"[MeSH Terms]) OR "space flight"[MeSH Terms]) | 20 |
| 117 | What is the minimum level of onboard medical skills / equipment / supplies for post ROSC medical management to justify on board CPR / defib? | ((((medical[All Fields] AND ("education"[Subheading] OR "education"[All Fields] OR "training"[All Fields] OR "education"[MeSH Terms] OR "training"[All Fields])) OR (medical[All Fields] AND skills[All Fields])) OR ("education, medical"[MeSH Terms] OR ("education"[All Fields] AND "medical"[All Fields]) OR "medical education"[All Fields] OR ("medical"[All Fields] AND "education"[All Fields]))) OR (("first aid"[MeSH Terms] OR ("first"[All Fields] AND "aid"[All Fields]) OR "first aid"[All Fields]) AND ("education"[Subheading] OR "education"[All Fields] OR "training"[All Fields] OR "education"[MeSH Terms] OR "training"[All Fields]))) AND ((((("weightlessness"[MeSH Terms] OR "weightlessness"[All Fields] OR "microgravity"[All Fields]) OR "weightlessness"[MeSH Terms]) OR ("weightlessness"[MeSH Terms] OR "weightlessness"[All Fields])) OR "weightlessness"[MeSH Terms]) OR "space flight"[MeSH Terms]) | 245 |
| 118 | What are the medical skill and training requirements to deliver cpr, defib and post-ROSC medical management? | ((((medical[All Fields] AND ("education"[Subheading] OR "education"[All Fields] OR "training"[All Fields] OR "education"[MeSH Terms] OR "training"[All Fields])) OR (medical[All Fields] AND skills[All Fields])) OR ("education, medical"[MeSH Terms] OR ("education"[All Fields] AND "medical"[All Fields]) OR "medical education"[All Fields] OR ("medical"[All Fields] AND "education"[All Fields]))) OR (("first aid"[MeSH Terms] OR ("first"[All Fields] AND "aid"[All Fields]) OR "first aid"[All Fields]) AND ("education"[Subheading] OR "education"[All Fields] OR "training"[All Fields] OR "education"[MeSH Terms] OR "training"[All Fields]))) AND ((((("weightlessness"[MeSH Terms] OR "weightlessness"[All Fields] OR "microgravity"[All Fields]) OR "weightlessness"[MeSH Terms]) OR ("weightlessness"[MeSH Terms] OR "weightlessness"[All Fields])) OR "weightlessness"[MeSH Terms]) OR "space flight"[MeSH Terms]) | 245 |
| 119 | What are the training / currency requirements during long duration spaceflight to ensure adequate CPR / defib / post-ROSC management?  🡪repitition | ((((medical[All Fields] AND ("education"[Subheading] OR "education"[All Fields] OR "training"[All Fields] OR "education"[MeSH Terms] OR "training"[All Fields])) OR (medical[All Fields] AND skills[All Fields])) OR ("education, medical"[MeSH Terms] OR ("education"[All Fields] AND "medical"[All Fields]) OR "medical education"[All Fields] OR ("medical"[All Fields] AND "education"[All Fields]))) OR (("first aid"[MeSH Terms] OR ("first"[All Fields] AND "aid"[All Fields]) OR "first aid"[All Fields]) AND ("education"[Subheading] OR "education"[All Fields] OR "training"[All Fields] OR "education"[MeSH Terms] OR "training"[All Fields]))) AND ((((("weightlessness"[MeSH Terms] OR "weightlessness"[All Fields] OR "microgravity"[All Fields]) OR "weightlessness"[MeSH Terms]) OR ("weightlessness"[MeSH Terms] OR "weightlessness"[All Fields])) OR "weightlessness"[MeSH Terms]) OR "space flight"[MeSH Terms]) | 245 |
| 120 | What are the most expected reversible causes for cardiac arrest in microgravity? | (("heart arrest"[MeSH Terms] OR ("heart"[All Fields] AND "arrest"[All Fields]) OR "heart arrest"[All Fields] OR ("cardiac"[All Fields] AND "arrest"[All Fields]) OR "cardiac arrest"[All Fields]) OR "heart arrest"[MeSH Terms]) AND ((((("weightlessness"[MeSH Terms] OR "weightlessness"[All Fields] OR "microgravity"[All Fields]) OR "weightlessness"[MeSH Terms]) OR ("weightlessness"[MeSH Terms] OR "weightlessness"[All Fields])) OR "weightlessness"[MeSH Terms]) OR "space flight"[MeSH Terms]) | 11 |
| 121 | Hypo-/hyperthermia: temperature regulation not working (Space vehicle or EVA suit) highly unlikely | (("hypothermia"[MeSH Terms] OR "hypothermia"[All Fields]) OR ("fever"[MeSH Terms] OR "fever"[All Fields] OR "hyperthermia"[All Fields])) AND ((((("weightlessness"[MeSH Terms] OR "weightlessness"[All Fields] OR "microgravity"[All Fields]) OR "weightlessness"[MeSH Terms]) OR ("weightlessness"[MeSH Terms] OR "weightlessness"[All Fields])) OR "weightlessness"[MeSH Terms]) OR "space flight"[MeSH Terms]) | 42 |
| 122 | Hypo-/hyperkalemia: incidence in space? Why should it be higher? Blood gas analysis for detection | (("hypokalaemia"[All Fields] OR "hypokalemia"[MeSH Terms] OR "hypokalemia"[All Fields]) OR ("hyperkalaemia"[All Fields] OR "hyperkalemia"[MeSH Terms] OR "hyperkalemia"[All Fields])) AND ((((("weightlessness"[MeSH Terms] OR "weightlessness"[All Fields] OR "microgravity"[All Fields]) OR "weightlessness"[MeSH Terms]) OR ("weightlessness"[MeSH Terms] OR "weightlessness"[All Fields])) OR "weightlessness"[MeSH Terms]) OR "space flight"[MeSH Terms]) | 4 |
| 123 | Hypovolemia/hemorrhage: Burn wound? Anaphylaxis? Sepsis? Injury while doing EVA? Incidence of injuries in space | ((((("hypovolaemia"[All Fields] OR "hypovolemia"[MeSH Terms] OR "hypovolemia"[All Fields]) OR ("haemorrhage"[All Fields] OR "hemorrhage"[MeSH Terms] OR "hemorrhage"[All Fields])) OR burning[All Fields]) OR combustion[All Fields]) OR ("sepsis"[MeSH Terms] OR "sepsis"[All Fields])) AND ((((("weightlessness"[MeSH Terms] OR "weightlessness"[All Fields] OR "microgravity"[All Fields]) OR "weightlessness"[MeSH Terms]) OR ("weightlessness"[MeSH Terms] OR "weightlessness"[All Fields])) OR "weightlessness"[MeSH Terms]) OR "space flight"[MeSH Terms]) | 170 |
| 124 | Hypoxia: oxygen system not working (space vehicle or EVA suit) incidence of serious events in the past? Drowning (malfunction of EVA suit)? | (("hypoxia"[MeSH Terms] OR "hypoxia"[All Fields]) OR ("drowning"[MeSH Terms] OR "drowning"[All Fields])) AND ((((("weightlessness"[MeSH Terms] OR "weightlessness"[All Fields] OR "microgravity"[All Fields]) OR "weightlessness"[MeSH Terms]) OR ("weightlessness"[MeSH Terms] OR "weightlessness"[All Fields])) OR "weightlessness"[MeSH Terms]) OR "space flight"[MeSH Terms]) | 149 |
| 125 | Intoxication: Possible toxins during space missions? No drugs/alcohol. Maybe operating materials/ chemicals? | (intoxication[All Fields] OR chemicals[All Fields]) AND ((((("weightlessness"[MeSH Terms] OR "weightlessness"[All Fields] OR "microgravity"[All Fields]) OR "weightlessness"[MeSH Terms]) OR ("weightlessness"[MeSH Terms] OR "weightlessness"[All Fields])) OR "weightlessness"[MeSH Terms]) OR "space flight"[MeSH Terms]) | 78 |
| 126 | Tension pneumothorax: incidence of spontaneous pneumothorax in space? Changing pressure levels? Young, athletic men? Penetrating chest trauma in space? blunt chest trauma in space? Association with barotrauma? | ((("pneumothorax"[MeSH Terms] OR "pneumothorax"[All Fields]) OR ("pneumothorax"[MeSH Terms] OR "pneumothorax"[All Fields] OR ("tension"[All Fields] AND "pneumothorax"[All Fields]) OR "tension pneumothorax"[All Fields])) OR ("thoracic injuries"[MeSH Terms] OR ("thoracic"[All Fields] AND "injuries"[All Fields]) OR "thoracic injuries"[All Fields] OR ("chest"[All Fields] AND "trauma"[All Fields]) OR "chest trauma"[All Fields])) AND ((((("weightlessness"[MeSH Terms] OR "weightlessness"[All Fields] OR "microgravity"[All Fields]) OR "weightlessness"[MeSH Terms]) OR ("weightlessness"[MeSH Terms] OR "weightlessness"[All Fields])) OR "weightlessness"[MeSH Terms]) OR "space flight"[MeSH Terms]) | 12 |
| 127 | Pulmonary embolism: incidence of spontaneous thrombosis/embolism in space? Probably higher incidence in immobile patients (affected by injury/infection/ other diseases) | ((((("weightlessness"[MeSH Terms] OR "weightlessness"[All Fields] OR "microgravity"[All Fields]) OR "weightlessness"[MeSH Terms]) OR ("weightlessness"[MeSH Terms] OR "weightlessness"[All Fields])) OR "weightlessness"[MeSH Terms]) OR "space flight"[MeSH Terms]) AND ((("embolism"[MeSH Terms] OR ("embolism"[MeSH Terms] OR "embolism"[All Fields])) OR "pulmonary embolism"[MeSH Terms]) OR ("thrombosis"[MeSH Terms] OR "thrombosis"[All Fields])) | 42 |
| 128 | Pericardial tamponade: See pneumothorax | ((((("weightlessness"[MeSH Terms] OR "weightlessness"[All Fields] OR "microgravity"[All Fields]) OR "weightlessness"[MeSH Terms]) OR ("weightlessness"[MeSH Terms] OR "weightlessness"[All Fields])) OR "weightlessness"[MeSH Terms]) OR "space flight"[MeSH Terms]) AND ("cardiac tamponade"[MeSH Terms] OR ("pericardium"[MeSH Terms] OR "pericardium"[All Fields] OR "pericardial"[All Fields])) | 2 |
| 129 | Should echocardiography/sonography be used during CPR in microgravity to identify potentially reversible causes for cardiac arrest? | (((((("critical care"[MeSH Terms] OR ("critical"[All Fields] AND "care"[All Fields]) OR "critical care"[All Fields]) OR ("emergencies"[MeSH Terms] OR "emergencies"[All Fields] OR "emergency"[All Fields])) OR ("cardiopulmonary resuscitation"[MeSH Terms] OR ("cardiopulmonary"[All Fields] AND "resuscitation"[All Fields]) OR "cardiopulmonary resuscitation"[All Fields] OR "cpr"[All Fields])) OR cardiopulmonary[All Fields]) OR ("resuscitation"[MeSH Terms] OR "resuscitation"[All Fields])) AND ((("ultrasonography"[MeSH Terms] OR ("ultrasonography"[MeSH Terms] OR "ultrasonography"[All Fields] OR "sonography"[All Fields])) OR "echocardiography"[MeSH Terms]) OR ("echocardiography"[MeSH Terms] OR "echocardiography"[All Fields]))) AND ((((("weightlessness"[MeSH Terms] OR "weightlessness"[All Fields] OR "microgravity"[All Fields]) OR "weightlessness"[MeSH Terms]) OR ("weightlessness"[MeSH Terms] OR "weightlessness"[All Fields])) OR "weightlessness"[MeSH Terms]) OR "space flight"[MeSH Terms]) | 26 |
| 130 | What is the incidence of ebullism/ severe decompression sickness in space mission? | (((((("weightlessness"[MeSH Terms] OR "weightlessness"[All Fields] OR "microgravity"[All Fields]) OR "weightlessness"[MeSH Terms]) OR ("weightlessness"[MeSH Terms] OR "weightlessness"[All Fields])) OR "weightlessness"[MeSH Terms]) OR "space flight"[MeSH Terms]) AND (ebullism[All Fields] OR ("decompression"[MeSH Terms] OR "decompression"[All Fields]))) AND ((((("critical care"[MeSH Terms] OR ("critical"[All Fields] AND "care"[All Fields]) OR "critical care"[All Fields]) OR ("emergencies"[MeSH Terms] OR "emergencies"[All Fields] OR "emergency"[All Fields])) OR ("cardiopulmonary resuscitation"[MeSH Terms] OR ("cardiopulmonary"[All Fields] AND "resuscitation"[All Fields]) OR "cardiopulmonary resuscitation"[All Fields] OR "cpr"[All Fields])) OR cardiopulmonary[All Fields]) OR ("resuscitation"[MeSH Terms] OR "resuscitation"[All Fields])) | 46 |
| 131 | What is the incidence of cardiac arrhythmia during space mission? | ((("arrhythmias, cardiac"[MeSH Terms] OR ("arrhythmias"[All Fields] AND "cardiac"[All Fields]) OR "cardiac arrhythmias"[All Fields] OR "arrhythmia"[All Fields]) OR ("tachycardia"[MeSH Terms] OR "tachycardia"[All Fields])) OR ("bradycardia"[MeSH Terms] OR "bradycardia"[All Fields])) AND ((((("weightlessness"[MeSH Terms] OR "weightlessness"[All Fields] OR "microgravity"[All Fields]) OR "weightlessness"[MeSH Terms]) OR ("weightlessness"[MeSH Terms] OR "weightlessness"[All Fields])) OR "weightlessness"[MeSH Terms]) OR "space flight"[MeSH Terms]) | 143 |
| 132 | How does medical preflight screening change the incidence of cardiac arrests in the screened population on space missions? | ((((preflight[All Fields] AND ("diagnosis"[Subheading] OR "diagnosis"[All Fields] OR "screening"[All Fields] OR "mass screening"[MeSH Terms] OR ("mass"[All Fields] AND "screening"[All Fields]) OR "mass screening"[All Fields] OR "screening"[All Fields] OR "early detection of cancer"[MeSH Terms] OR ("early"[All Fields] AND "detection"[All Fields] AND "cancer"[All Fields]) OR "early detection of cancer"[All Fields])) OR ("physical examination"[MeSH Terms] OR ("physical"[All Fields] AND "examination"[All Fields]) OR "physical examination"[All Fields] OR "examination"[All Fields])) OR ("diagnosis"[Subheading] OR "diagnosis"[All Fields] OR "screening"[All Fields] OR "mass screening"[MeSH Terms] OR ("mass"[All Fields] AND "screening"[All Fields]) OR "mass screening"[All Fields] OR "screening"[All Fields] OR "early detection of cancer"[MeSH Terms] OR ("early"[All Fields] AND "detection"[All Fields] AND "cancer"[All Fields]) OR "early detection of cancer"[All Fields])) AND ((((("critical care"[MeSH Terms] OR ("critical"[All Fields] AND "care"[All Fields]) OR "critical care"[All Fields]) OR ("emergencies"[MeSH Terms] OR "emergencies"[All Fields] OR "emergency"[All Fields])) OR ("cardiopulmonary resuscitation"[MeSH Terms] OR ("cardiopulmonary"[All Fields] AND "resuscitation"[All Fields]) OR "cardiopulmonary resuscitation"[All Fields] OR "cpr"[All Fields])) OR cardiopulmonary[All Fields]) OR ("resuscitation"[MeSH Terms] OR "resuscitation"[All Fields]))) AND ((((("weightlessness"[MeSH Terms] OR "weightlessness"[All Fields] OR "microgravity"[All Fields]) OR "weightlessness"[MeSH Terms]) OR ("weightlessness"[MeSH Terms] OR "weightlessness"[All Fields])) OR "weightlessness"[MeSH Terms]) OR "space flight"[MeSH Terms]) | 207 |
| 133 | Do changes in cardiac cellular physiology in microgravity alter likelihood of arrhythmias or cardiac arrest or likelihood of successful resuscitation? | ((("physiology"[Subheading] OR "physiology"[All Fields] OR "physiology"[MeSH Terms]) OR ("cells"[MeSH Terms] OR "cells"[All Fields] OR "cellular"[All Fields])) AND ((("arrhythmias, cardiac"[MeSH Terms] OR ("arrhythmias"[All Fields] AND "cardiac"[All Fields]) OR "cardiac arrhythmias"[All Fields] OR "arrhythmia"[All Fields]) OR ("tachycardia"[MeSH Terms] OR "tachycardia"[All Fields])) OR ("bradycardia"[MeSH Terms] OR "bradycardia"[All Fields]))) AND ((((("weightlessness"[MeSH Terms] OR "weightlessness"[All Fields] OR "microgravity"[All Fields]) OR "weightlessness"[MeSH Terms]) OR ("weightlessness"[MeSH Terms] OR "weightlessness"[All Fields])) OR "weightlessness"[MeSH Terms]) OR "space flight"[MeSH Terms]) | 108 |
| 134 | Incidence of spacesuit or habitat rapid or complete depressurization? | ((depressurization[All Fields] OR (("pressure"[MeSH Terms] OR "pressure"[All Fields]) AND loss[All Fields])) OR (("pressure"[MeSH Terms] OR "pressure"[All Fields]) AND drop[All Fields])) AND ((((("weightlessness"[MeSH Terms] OR "weightlessness"[All Fields] OR "microgravity"[All Fields]) OR "weightlessness"[MeSH Terms]) OR ("weightlessness"[MeSH Terms] OR "weightlessness"[All Fields])) OR "weightlessness"[MeSH Terms]) OR "space flight"[MeSH Terms]) | 176 |
